# Benzofuran compound from *Sophora tonkinensis Gagnep* suppresses nasopharyngeal carcinoma growth via PI3K/AKT/mTOR pathway: evidence from molecular dynamics simulation and *in vitro* experiments

**DOI:** 10.3389/fphar.2026.1778697

**Published:** 2026-04-23

**Authors:** Peiying Wu, Xiaomei Gong, Wenyu Zhang, Dandan Mo, Lili He, Zhijun Song, Chunli Ou, Meng Li, Xiaolei Zhou, Xingyu Xiao, Xianghua Xia, Shuo Wang

**Affiliations:** 1 National Engineering Research Center of Southwest Endangered Medicinal Resources Development, Guangxi Botanical Garden of Medicinal Plants, Nanning, China; 2 Department of Pharmacy, Guangxi Medical University Cancer Hospital, Nanning, China; 3 Guangxi Key Laboratory of Medicinal Resources Protection and Genetic Improvement, Guangxi Botanical Garden of Medicinal Plants, Nanning, China

**Keywords:** apoptosis, benzofuran compound, nasopharyngeal carcinoma, PI3K/AKT/ mTOR/signaling pathway, Sophora tonkinensis Gagnep

## Abstract

**Introduction:**

Shandougen (SDG), the dried root and rhizome of *Sophora tonkinensis Gagnep.*, has been historically documented in Ben Cao Qiu Zhen for its properties of “clearing heat, detoxifying, reducing swelling, and relieving throat disorders.” It is highly valued as a primary herb for relieving throat swelling and abscesses. SDG-based formulas, such as “Bi Yan Ling Tablets” and the “Bi’ai Formula,” have shown definite efficacy against heat-toxin accumulation type nasopharyngeal carcinoma (NPC). Despite this, the specific active monomer in SDG inhibiting NPC remains unclear, warranting further investigation.

**Objective:**

This study aimed to identify the bioactive monomers underlying SDG’s anti-NPC efficacy and elucidate their molecular mechanisms.

**Methods:**

Guided by HPLC fingerprinting, targeted isolation and purification of components from the active fraction were performed. 13 compounds were screened for activity, with benzofuran compound 2-(2′,4′-dihydroxyphenyl)-5,6-methylenedioxybenzofuran (ABF) showing the most potent *in vitro* effects. Molecular dynamics (MD) simulations were employed to evaluate the binding affinity and stability of ABF with the target protein. The anti-proliferative mechanisms of ABF were investigated in cells, and its anti-tumor capacity was evaluated using a nude mouse xenograft model.

**Results:**

ABF exhibited stronger anti-proliferative activity against CNE1 and CNE2 cells compared to SDG extract, with a 10-fold lower 48-h IC_50_ value. ABF inhibits the invasion and metastasis of NPC cells, which is associated with S-phase cell cycle arrest and the downregulation of cyclin E1 and CDK2 proteins. Apoptosis induction by ABF was associated with increased caspase family proteins and lowered the Bcl-2/Bax ratio. PCR and Western blot analyses confirmed that ABF is involved in the downregulation of the PI3K/AKT/mTOR pathway, inhibiting NPC cell growth. Additionally, in a nude mouse xenograft model, ABF treatment at high doses achieved tumor inhibition rates of 46.59% and 45.71%, respectively,and the expression of the tumor proliferation marker KI67 was significantly reduced.

**Conclusion:**

ABF demonstrates promising anti-NPC potential, providing scientific evidence for the traditional anti-cancer use of SDG. This molecule may serve as a natural lead compound for NPC growth inhibitors.

## Graphical Abstract

**Figure:**
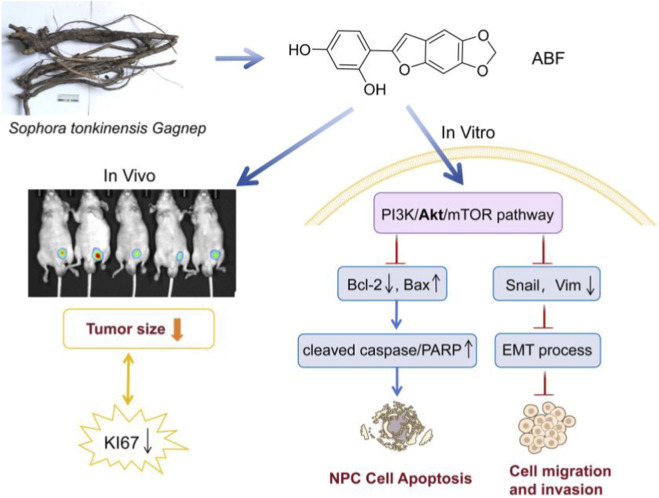

## 1 Introduction

Shandougen (SDG), also known as Rangduba in Zhuang medicine, is the dried root and rhizome of *Sophora tonkinensis Gagnep*., belonging to the genus Sophora (Fabaceae). It is mainly distributed in the karst regions of southwest China, extending to northern Vietnam. As a representative medicinal plant with cross-border distribution in Southeast Asia, it exhibits both phytochemical diversity and significant pharmacological potential, serving as a key species that links medicinal plant research and application between Vietnam and China. In the traditional medicine systems of both China and Vietnam, Shandougen has been used for centuries, mainly for clearing heat, detoxifying, soothing sore throat, and reducing swelling. Modern research continues to validate and expand its traditional uses (see Supplementary Material Table 1). Studies have shown that Shandougen is rich in various secondary metabolites, including alkaloids, flavonoids, benzofurans, polysaccharides, and triterpenoid saponins (Zeng et al., 2025). This complex chemical composition is a core prerequisite for the discovery of lead compounds.

Since the Ming Dynasty’s *Compendium of Materia Medica*, it has been recorded that the variety produced in Guangxi is of the finest quality, a reputation that continues to this day. Its main producing areas are located in the karst mountainous regions of Hechi and Baise in Guangxi, China. As one of the “Gui Shi Wei (Ten geo-authentic crude drugs of Guangxi)” and a key herb in Zhuang medicine, it has been recognized for its effects in “clearing heat, detoxifying, reducing swelling, and relieving throat disorder” as documented in Ben Cao Qiu Zhen (Gongxiu, 1987), where it is highly valued as a primary herb for relieving throat swelling and abscesses. According to traditional Zhuang medicine theory (Yan et al., 2017), nasopharyngeal carcinoma (NPC) falls under the categories of “Shi Rong” (loss of nourishment) and “Bi Ju” (nasal pustule), with the core pathogenesis being internal accumulation of heat-toxicity, stagnation of heat, and phlegm-stasis binding. Therefore, herbs that “clear heat and resolve abscess” are considered key to interrupting this pathological process (Zhang et al., 2020). Clinically, compound formulations based on SDG, such as “Biyanling Tablets” and the “Bi’ai Formula,” have demonstrated definite efficacy in treating heat-toxicity accumulation-type NPC (Lu, 2012). The extract of SDG has been confirmed to exhibit significant anti-NPC activity (Wang et al., 2021; Zeng et al., 2025). Its constituents, including flavonoids, benzofurans, alkaloids, triterpenoids, and polysaccharides, can inhibit tumor cell growth, differentiation, and migration, while promoting apoptosis through multiple targets and pathways. Based on reported anti-NPC cellular activity of SDG extracts, we believe it holds significant development potential. However, the complex composition of the extract and the inherent toxicity of its total alkaloids pose challenges for passing rigorous preclinical studies. Therefore, we focused on screening the major anti-NPC active components from the chloroform extract of SDG and validating their efficacy and safety.

Using silica gel, gel, and ODS column chromatography guided by HPLC fingerprint analysis, we isolated and purified 13 compounds from the anti-NPC active fraction of SDG. Through activity screening, benzofuran derivative 2-(2′,4′-dihydroxyphenyl)-5,6-methylenedioxybenzofuran (ABF, an abbreviation following (Jang et al., 2015) nomenclature) demonstrated the most potent efficacy *in vitro*. Comparison the monomeric ABF with the SDG extract revealed significantly superior anti-NPC activity for ABF. Its IC_50_ values against CNE1 and CNE2 cells were 4.53 ± 1.03 μg/mL and 3.93 ± 1.27 μg/mL, respectively, nearly 10-fold lower than those of the extract (IC_50_: 46.64 ± 0.38 μg/mL and 31.40 ± 0.66 μg/mL) (Wang et al., 2021), indicating stronger anti-proliferative activity. Our investigation into the anti-NPC activity of ABF, an active ingredient of SDG, essentially constitutes a modern molecular validation of the herb’s traditional efficacy: its described function of “clearing heat and detoxifying” corresponds to ABF’s observed anti-proliferative and pro-apoptotic effects (eliminating “heat-toxin” equates to inhibiting tumor cells); while its traditional use for” reducing swelling and relieving throat discomfort” is consistent with ABF’s ability to suppress migration and invasion (alleviating “swelling and pain” by blocking tumor metastasis). This provides molecular-level evidence linking its traditional “reducing swelling and relieving throat disorders” efficacy to specific biological effects (inhibition of NPC), demonstrating the rationale of “guiding modern research on Chinese medicine with traditional theories”.

Benzofuran compounds have been previously reported as drug lead molecules (Abbas and Dawood, 2023; Arce-Ramos et al., 2023). While the pharmacokinetic properties of ABF have been reported previously (Jang et al., 2015), its antitumor activity remains unexplored, and its inhibitory effect on NPC has not been sufficiently validated. Therefore, further evaluation of ABF’s antitumor efficacy *in vitro* and *in vivo*, and elucidation of its mechanism of action, are necessary to clarify its potential as a natural anti-NPC agent or lead compound and to promote the development of SDG.

## 2 Materials and methods

### 2.1 Preparation and characterization of ABF

SDG was collected from Jingxi City, Guangxi Province, and authenticated as genuine by Professor Zhou Xiaolei and is stored at Guangxi Botanical Garden of Medicinal Plants (voucher specimen:Z20220706005). 10 kg SDG powder (pulverized through a 10-mesh sieve) was macerated 3 times with 75% ethanol at a solid-to-liquid ratio of 1:10 (m/v). The ethanol was then recovered using a rotary evaporator (Ailang Instrument CCA-1112, Shanghai, China), and the aqueous phases were combined. The combined aqueous phase was sequentially subjected to liquid-liquid extraction with equal volumes of petroleum ether, chloroform, ethyl acetate, and n-butanol (each solvent used 3 times), yielding four solvent-extracted fractions. After concentration by rotary evaporation to remove the solvents, 1.10 kg of the chloroform extract was obtained. The chloroform extract was subjected to column chromatography over silica gel (100–200 mesh) and eluted with a gradient of petroleum ether–chloroform (80:1 → 0:1, v/v) followed by chloroform–methanol (1:0 → 1:1, v/v), yielding 10 fractions (Fr. A–Fr. J). HPLC analysis indicated that the target compounds were primarily concentrated in Fr. F. Fraction Fr. F was further separated using a preparative liquid chromatography system (VARIAN SD2) coupled with a medium-pressure Flash column (Silica, 20–35 μm, 120 Å) and eluted with a gradient of methanol–water (50:50 → 100:0, v/v), resulting in 12 subfractions (Fr. F-1 – Fr. F-12). Subfraction Fr. F-11 was purified by Sephadex LH-20 gel column chromatography (eluent: chloroform–methanol = 1:1, v/v) and subsequently on an ODS column (5.0 × 60 mm) with a gradient of methanol–water (20:80 → 100:0, v/v), yielding 89 mg of a white powder (Batch No. A-01). The structure of the obtained compound was elucidated using a high-resolution NMR spectrometer (Bruker, Germany) operating at 600 MHz for ^1^H and 150 MHz for ^13^C NMR measurements, with DMSO-d_6_ as the solvent.

**FIGURE 1:**
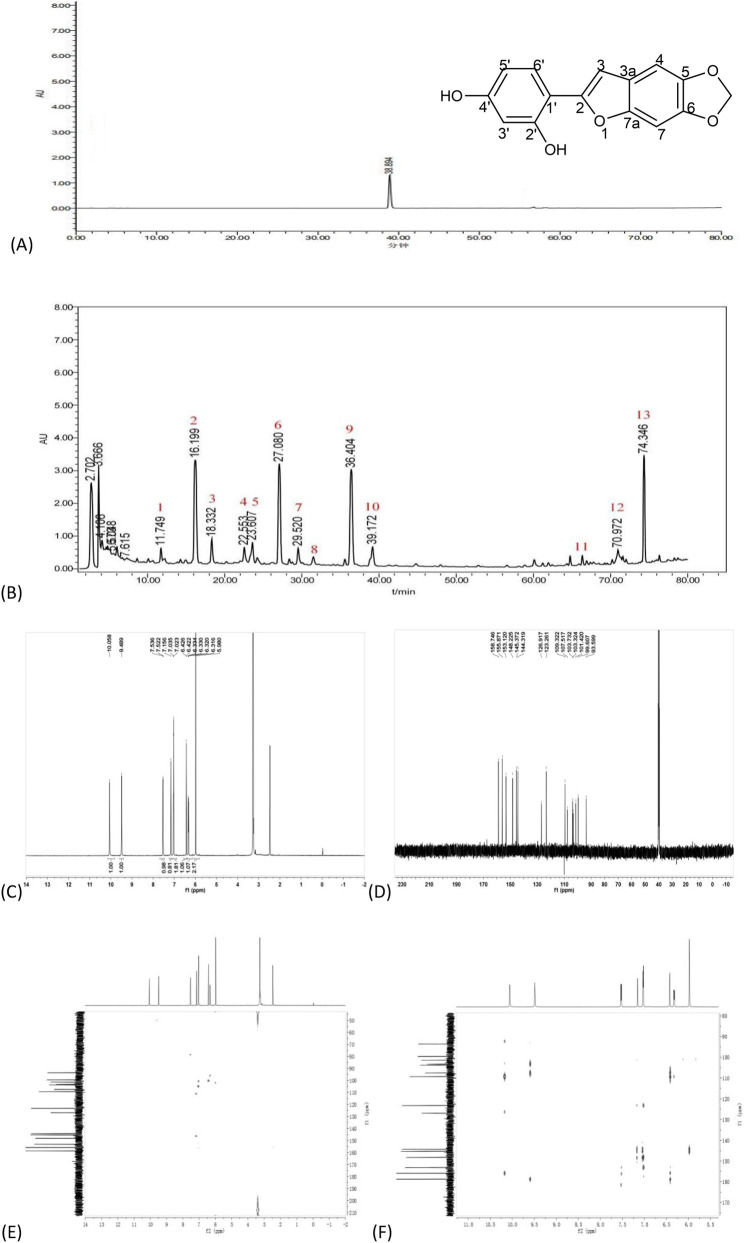
Position of ABF in the HPLC chromatogram of the SDG extract and NMR information. **(A)** HPLC chromatogram of compound ABF. t_min_ = 38.89 min **(B)** HPLC chromatogram of SDG chloroform extract. Peak 10 corresponds to the retention time of ABF. Reproduced from (Wang et al., 2021), Figure 1d, under CC BY‐NC‐ND license: 6251300582449. **(C)** ^1^H-NMR spectrum of ABF. **(D)** ^13^C-NMR spectrum of ABF. **(E)** HMQC spectrum of ABF. **(F)** HMBC spectrum of ABF.

The obtained compound was dissolved in methanol to prepare a 20 μg/mL reference solution. Its purity was determined by HPLC (Agilent 1260) - UV detection at 205 nm. Based on peak area normalization (Figure 1A), the purity was calculated to be 98.20%. The SDG chloroform extract (2g) was dissolved in methanol and volumetrically adjusted to 10 mL as the test sample solution. Agilent 1260 Infinity II Prime LC system was used with a Waters Sunfire C18 column (4.6 × 250 mm, 5 μm) under the following parameters: 1.0 mL/min, 205 nm, 30 °C, and 10 μL injection. The water (A)/acetonitrile (B) gradient was: 0–5 min, 25% B; 5–55 min, 25%–50% B; 55–80 min, 50%–95% B.

### 2.2 MD simulation and CYP metabolism prediction

Protein structures in PDB format (1E8X for PI3K, 3CQW for AKT1, and 4JSV for mTOR) were obtained from the RCSB PDB database. The SDF format of ABF was downloaded from PubChem and imported into Maestro for receptor and ligand preprocessing. The prepared ligands and receptors were subjected to molecular docking using Glide, and the results were visualized with PyMOL. MD simulations were performed using Amber 24 with ff14SB and phossa force fields; the ligand force field was gaff. The TIP3P water model was used for solvation, and 0.15 M counterions (Na^+^ and Cl^−^) were added to neutralize the system. After pre-equilibration at 310K (37 °C), MD simulations were conducted for the three systems under isothermal-isobaric conditions with a time step of 2 fs, saving a trajectory frame every 4,000 steps. The binding free energy between the ligand and protein was calculated using the MM/GBSA method. Multi-organ toxicity of ABF was predicted using ADMETlab 3.0, while metabolic sites and isoform selectivity were predicted using MetaSite software.

### 2.3 Cell viability measurement

The NPC cell lines CNE1 and CNE2 (sample codes: 20171106-02, 20171012-01) and the human normal pharyngeal cell line NP69 were purchased from Shanghai Enzyme Research Biotechnology Co., Ltd. Cell viability was assessed using a CCK-8 kit. To assess compound efficacy, CNE1 or CNE2 cells were plated at 5 × 10^4^ cells/mL and then treated with individual compounds (1-13) at 10 μg/mL. After 48 h, the inhibition rate was evaluated. Next, cells were incubated with ABF across a concentration range of 1–10 μg/mL for 24, 48, and 72 h, respectively, with cisplatin (7.5 μg/mL) used as the positive control. IC_50_ calculated from OD_450_ nm readings (BioTek ELx800, United States) via GraphPad Prism 9.0.

### 2.4 Colony formation, migration and invasion assay

A single batch of ABF (Batch No. A-01) was used for all biological assays. CNE1 and CNE2 cells were treated with media containing ABF (2, 4 μg/mL). The experimental procedures for colony formation, migration, and invasion assays were consistent with the reported methods (Gong et al., 2024). Cell images were captured using an Olympus IX73P2F microscope (Tokyo, Japan). The scratch wound area and the number of cells that migrated through the transwel were quantified using ImageJ software.

### 2.5 Cell apoptosis and cycle analysis

Following seeding in a dish at 2 × 10^6^ cells/mL, NPC cells were exposed to ABF for 24, 48, and 72 h. Analysis was performed by flow cytometry (EMD Millipore guava easyCell Accuri C6) using: Annexin V-FITC kit (KeyGEN BioTECH, KGA105-KGA108) for apoptosis. Cell cycle detection kit (KeyGEN, KGA512) for cell cycle distribution. Data were analyzed using FlowJo software.

### 2.6 Western blot analysis

NPC cells treated with ABF (μg/mL) for 24 and 48 h were lysed using RIPA buffer. After sonication and centrifugation, the supernatant protein concentration was quantified by BCA assay kit (Beyotime Biotechnology, P0012S). Proteins were separated on SurePAGE Bis-Tris gels, transferred to PVDF membranes, blocked, and incubated overnight with primary antibodies (antibody information used in Supplementary Table S2). Following washes, the membranes were probed with a secondary antibody. Images were captured using a GE imaging system (Amersham Imager 680) and quantified with ImageJ software.

### 2.7 RT-PCR assay

Total RNA was extracted from ABF-treated cells using an RNA extraction kit (Promega, LS1040), and the RNA concentration and purity were determined. Subsequently, reverse transcription and amplification were performed using the 5X All-In-One RT MasterMix kit (ABM, G492) and a PCR thermal cycler (Roche, LightCycler 96). Gene expression was normalized to GAPDH and analyzed by the 2^–ΔΔCt method. See Supplementary Material (Supplementary Table S3) for the primer sequences used.

### 2.8 Animals and treatment

The pLV [Exp]-Puro-EF1A > Luciferase plasmid was transfected into 293T cells using liposome-based methods to package lentiviruses. After 48 h of transfection, the supernatant was collected, concentrated, and purified to determine the viral titer. CNE1/CNE2 cells were infected at an MOI of 50 with the addition of Polybrene. After 48 h, puromycin was used to select positive clones. Single positive clones were picked and expanded. Luciferase activity was validated *in vitro* by detecting luciferase activity, and the expression intensity of the reporter gene was measured to confirm stability, resulting in the generation of ^LUC−^CNE1/CNE2 stable cell lines.

The successfully constructed ^LUC−^CNE1/CNE2 cells were adjusted to a concentration of 1 × 10^7^ cells/mL. Following subcutaneous inoculation of 100 μL cell suspension in the groin of 30 nude mice (18–22g), successful model establishment was confirmed by an *in vivo* imaging system upon achieving a tumor fluorescence value of ≥1 × 10^7^ photons/second/cm^2^/steradian in all mice. A randomized block design was then performed to assign mice to different treatment groups. Briefly, the 30 mice with successfully established models were ranked according to their tumor fluorescence values and divided into 6 blocks, with 5 mice having similar fluorescence values in each block. Mice within each block were then randomly assigned to different groups (5 group, n = 6/group) using SPSS software. This ensured comparable baseline tumor sizes across all groups before treatment. The investigators performing drug administration and tumor measurement were blinded to the group assignments throughout the experiment. The mice were divided into a control group (0.5% carboxymethyl cellulose), a positive control group (cisplatin 5 mg/kg/d), and three ABF dose groups (20 mg/kg/d, 10 mg/kg/d, 5 mg/kg/d). Intraperitoneal injections were administered once weekly for 3 weeks. Tumor fluorescence values were monitored every 3 days, and the tumor inhibition rate (%) = (1 – T/C) × 100% (T and C: mean fluorescence value of treatment and control groups).

Tumor sections from each group were processed for KI67 immunohistochemistry. The procedure included deparaffinization, antigen retrieval with sodium citrate buffer, inactivation of catalase with hydrogen peroxidase, and blocking of nonspecific antigen sites. KI67 antibody was added and incubated overnight at 4 °C. After rewarming, a secondary antibody was applied and incubated at room temperature for 50 min. Following PBS washing, DAB development was performed, and the sections were re-dyed with hematoxylin, dehydrated, mounted, and examined under a microscope. After whole-slide scanning at low magnification (×40, ×100) to identify positive cell hotspots,the Ki67 index =(number of positive cells/number of tumor cells) * 100%.

### 2.9 Statistical analysis

All cell experiments were performed in triplicate, and representative results are shown. Statistical significance between groups was evaluated using Student’s t-test or one-way ANOVA. Data analysis was performed using SPSS 23.0 and GraphPad Prism 8.3.0. Data are presented as mean ± SD, with error bars representing SD. The criteria for statistical significance are as follows: *p < 0.05, **p < 0.01.

## 3 Results

### 3.1 Characterization of ABF

Guided by the HPLC fingerprint of the SDG chloroform extract (the active fraction against NPC), 13 compounds were isolated and purified. Among these, compound 10, which exhibited prominent activity, was obtained as white crystals (Figures 1A,B). ESI-MS displayed a peak at m/z 269.0447 [M-H]^-^, and structural analysis by ^1^H-NMR and ^13^C-NMR data revealed the molecular formula to be C_15_H_10_O_5_.

^1^H-NMR (600 MHz, DMSO-d_6_) δ_H_ ppm: 10.06 (1H) suggested a 2′-OH, and δ_H_ 9.49 (1H) indicated a 4′-OH. Signals at δ_H_ 6.43 (1H, d, J = 2.4 Hz), δ_H_ 6.33 (1H, dd, J = 2.4, 8.4 Hz), and δ_H_ 7.53 (1H, d, J = 8.4 Hz) were attributed to protons on a benzene ring. Based on the coupling constants, δ_H_ 6.33 (1H, dd, J = 2.4, 8.4 Hz) and δ_H_ 6.43 (1H, d, J = 2.4 Hz) were meta-coupled protons, while δ_H_ 6.33 and δ_H_ 7.53 (1H, d, J = 8.4 Hz) were ortho-coupled. Singlets at δ_H_ 7.04 (1H, s, H-4) and δ_H_ 7.16 (1H, s, H-7) were assigned to aromatic protons. ^13^C-NMR (150 MHz, DMSO-d_6_) δ_C_: δ_C_ 145.4 (C-5) and δ_C_ 144.3 (C-6) were adjacent to δ_C_ 101.4 (-OCH_2_O-). The data (Table.1) agreed with those from previous literature (McKittrick et al., 1982; Yoshiaki et al., 1999). Therefore, compound 10 was identified as 2-(2′,4′-dihydroxyphenyl)-5,6-methylenedioxybenzofuran (Figures 1C–F).

**TABLE 1:** ^1^H-NMR and^13^C-NMR data of 2-(2′,4′-dihidroxyphenyl)-5,6-methylenedioxybenzofuran.

Position	*δ* ^1^H- NMR (ppm)	Position	*δ* ^13^C-NMR (ppm)
​	​	2	153.1
H-3	7.02 (s)	3	103.7
​	​	3a	123.3
H-4	7.04 (s)	4	99.6
​	​	5	145.4
​	​	6	144.3
H-7	7.16(s)	7	93.6
​	​	7a	148.2
​	​	1′	109.3
2′-OH	10.06(s)	2′	155.9
H-3′	6.43 (d, *J* = 2.4)	3′	103.3
4′-OH	9.49 (s)	4′	158.7
H-5′	6.33 (dd, *J* = 2.4, 8.4)	5′	107.5
H-6′	7.53 (d, *J* = 8.4)	6′	126.9
-OCH_2_O-	5.98(s)	-OCH_2_O-	101.4

### 3.2 ABF inhibits the proliferation, migration, and invasion of NPC cells *in vitro*

**FIGURE 2:**
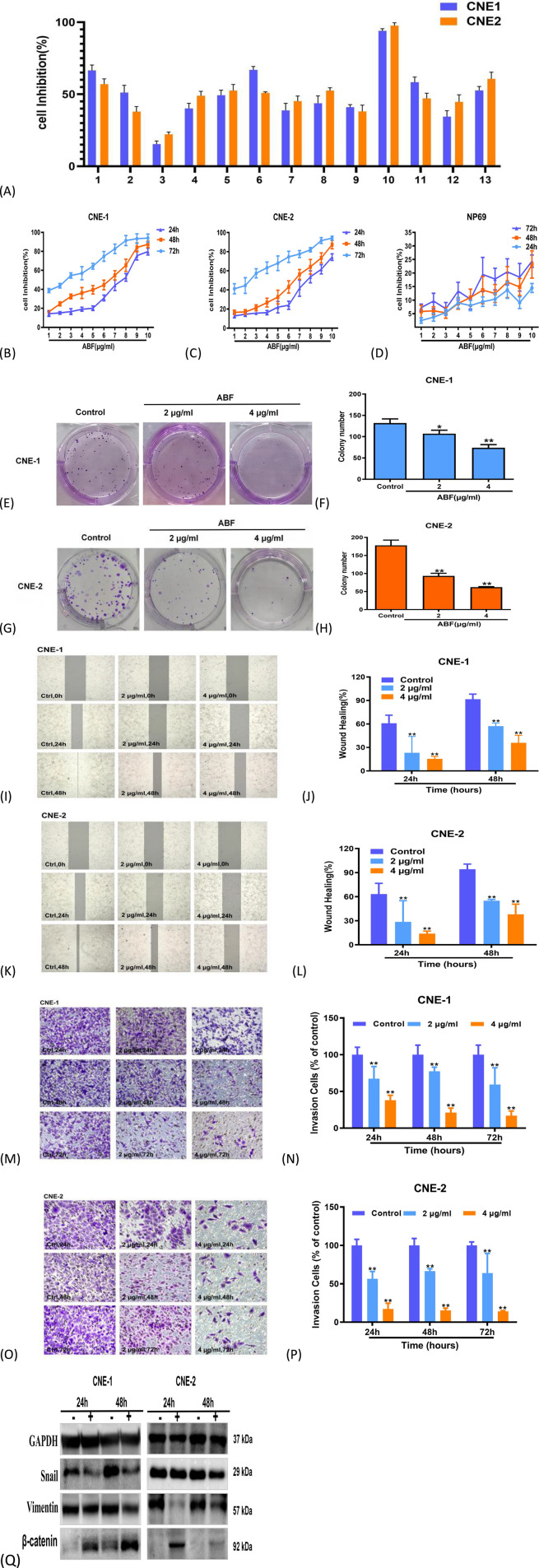
Effects of ABF on NPC Cell Activity. **(A)** Inhibition rates of compounds 1-13 (10 μg/mL) for 48 h **(B–D)** Cell viability of CNE1, CNE2, and NP69 cells treated with different doses of ABF. **(E–H)** Reduction in the number of cell colonies in ABF-treated groups. **(I–L)** Smaller wound healing areas in ABF-treated groups, indicating inhibited cell migration capability. Scale bar, 50 μm. **(M–P)** Fewer stained cells in ABF-treated groups, demonstrating decreased cell invasion capability. Scale bar, 100 μm. **(Q)** Effects of ABF on EMT marker expression. Mean ± SD, n = 3/group, Student’s t-test, *p < 0.05, **p < 0.01 vs. control group.

In the initial activity screening of 13 compounds, ABF demonstrated the most potent anti-proliferative activity against two NPC cell lines (Figure 2A). Our previous study showed that the chloroform extract treated CNE1 and CNE2 cells for 48 h with IC_50_ values of 46.64 ± 0.38 μg/mL and 31.40 ± 0.66 μg/mL, respectively. The results (Figures 2B–D) showed that the 48 h IC_50_ values of ABF in CNE1 and CNE2 cells were 4.53 ± 1.03 μg/mL and 3.93 ± 1.27 μg/mL, respectively, whereas the IC_50_ in normal nasopharyngeal epithelial NP69 cells was 58.30 ± 4.20 μg/mL. A selectivity index (SI) > 10 is generally regarded as desirable for lead compounds in early anticancer drug development. The SI values of ABF were 14.8 for CNE1 and 12.9 for CNE2. Furthermore, compared with the total extract, the effective concentration of ABF monomer was reduced by approximately 10-fold, and ABF showed no obvious toxicity toward NP69 cells at its IC_50_ concentration, indicating a favorable safety window.

Subsequently, after 8 days of drug administration to NPC cells, compared with the blank control group, the number of cell colonies in the ABF dose groups was significantly reduced (Figures 2E–H). In the scratch assay, at the 48-h mark, the wound in the blank control group had completely healed due to NPC cell migration without drug interference, whereas the wound healing was significantly reduced in ABF-treated NPC cells (Figures 2I–L). In the Transwell chamber assay (Figures 2M–P), at 72 h, the rates of cells penetrating the chamber in the 4 μg/mL ABF dose group were 17.04% and 14.06% for CNE1 and CNE2, respectively, demonstrating a marked reduction compared to the control group. The number of ABF-treated cells penetrating the Transwell chamber basement membrane was markedly decreased. These results indicate that ABF can significantly inhibit the proliferation of NPC cells, as well as reduce their migration and invasion capabilities.

Given the role of Epithelial-mesenchymal transition (EMT) in cancer metastasis, we measured EMT marker expression (β-Catenin, Vimentin, Snail) to assess ABF’s effect on NPC cell migration and invasion. It was found that after 48 h of ABF treatment, the expression of Snail and Vimentin was downregulated in NPC cells, while the expression of β-catenin was upregulated (Figure 2Q). We speculate that ABF can inhibit the biological process of EMT in NPC cells, thereby reducing their invasion and migration capabilities.

### 3.3 ABF treatment induced S-phase arrest in NPC cells

**FIGURE 3:**
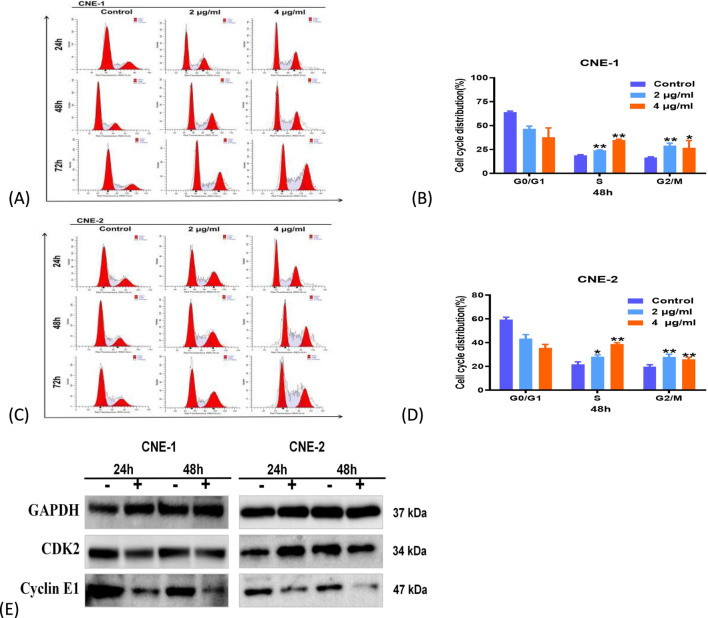
ABF arrests cell cycle progression of 2 cells. **(A–D)** Cell cycle distribution profiles in response to ABF treatment over 24, 48, and 72 h. Mean ± SD, n = 5/group, Student’s t-test, *p < 0.05, **p < 0.01 vs. control group. **(E)** Effects of ABF on S-phase-associated proteins in NPC cells.

Since cell proliferation is driven by the cell cycle, we employed flow cytometry to monitor it. Following 48 h of ABF treatment, both CNE1 and CNE-2 cells demonstrated an increase in the proportion of cells in the S phase (Figures 3A–D, ^**^ *P* < 0.01). Meanwhile, the expression of the S-phase regulatory proteins Cyclin E1 and CDK2 was downregulated (Figure 3E), indicating that cells were mainly arrested at this stage. The S phase is the stage of DNA replication and synthesis, and the observed arrest suggests that ABF delays cell proliferation by interfering with this critical process.

### 3.4 ABF elicits apoptosis in NPC cells by disrupting the Bcl-2/Bax balance

**FIGURE 4:**
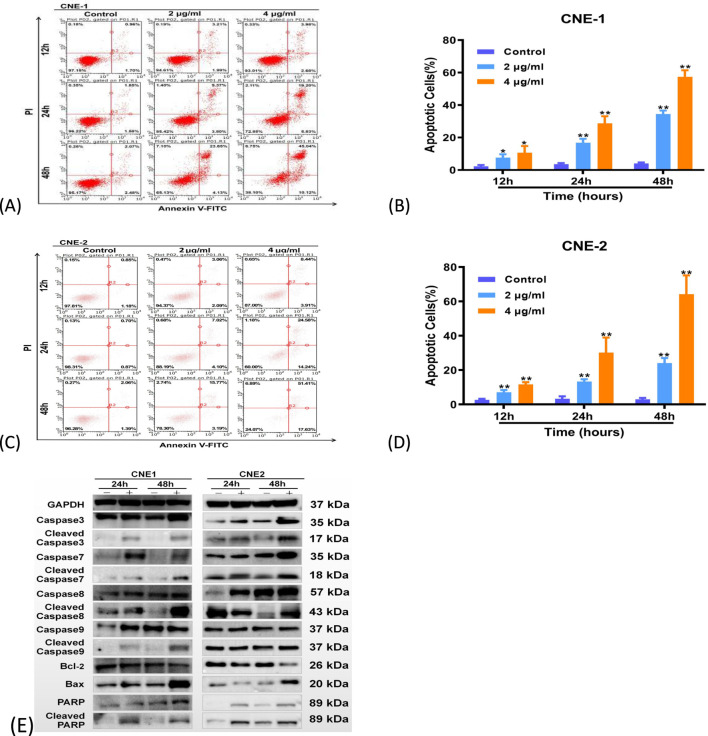
ABF triggers apoptosis in NPC cells. **(A–D)** Annexin V-FITC/PI double staining revealed that the proportion of apoptotic cells increased in the ABF-treated groups. **(E)** The levels of apoptosis marker proteins from the caspase family and Bax/Bcl-2 were altered following ABF treatment. Mean ± SD, n = 5/group, Student’s t-test, *p < 0.05, **p < 0.01 vs. control group.

The pro-apoptotic effect of ABF on NPC cells was investigated by measuring the apoptosis rate with Annexin V/PI staining. It was found that as the drug concentration and treatment time increased, the number of ABF-induced apoptotic NPC cells increased, and the apoptosis rate rose (Figures 4A–D). After 48 h of 4 mg/mL drug treatment, CNE-1 cells showed 10.12% early apoptosis, 45.04% late apoptosis, with a total apoptotic rate of 55.16%. Under the same conditions, CNE-2 cells had 17.63% early apoptosis and 51.41% late apoptosis, corresponding to a total apoptotic rate of 69.04% (P < 0.05, P < 0.01). Even when treated with the IC_50_ concentration, the apoptosis rate exceeded 50%, which corresponded with the CCK-8 results, indicating significant apoptosis. To further confirm ABF-induced apoptosis, we examined the Caspase protease family, which regulates apoptosis. The results showed (Figure 4E) that after ABF treatment, the pro-apoptotic protein Bax increased, while the anti-apoptotic protein Bcl-2 decreased. Furthermore, ABF upregulated the expression of Cleaved Caspase-3, -7, -8, -9, and Cleaved PARP, thereby inducing apoptosis in NPC cells.

### 3.5 Molecular simulation and metabolic prediction of ABF targeting the PI3K/Akt/mTOR pathway

The aberrant activation of the PI3K/Akt/mTOR pathway is closely associated with the malignant progression of nasopharyngeal carcinoma. It serves not only as an “engine” for tumor growth but also as a key biomarker of disease aggressiveness (Li et al., 2020). Therefore, we performed molecular docking and MD simulations of ABF with these three target proteins,and comprehensively evaluated its binding affinity and stability using root-mean-square deviation (RMSD), radius of gyration (Rg), and ligand–protein distance analysis.

**FIGURE 5:**
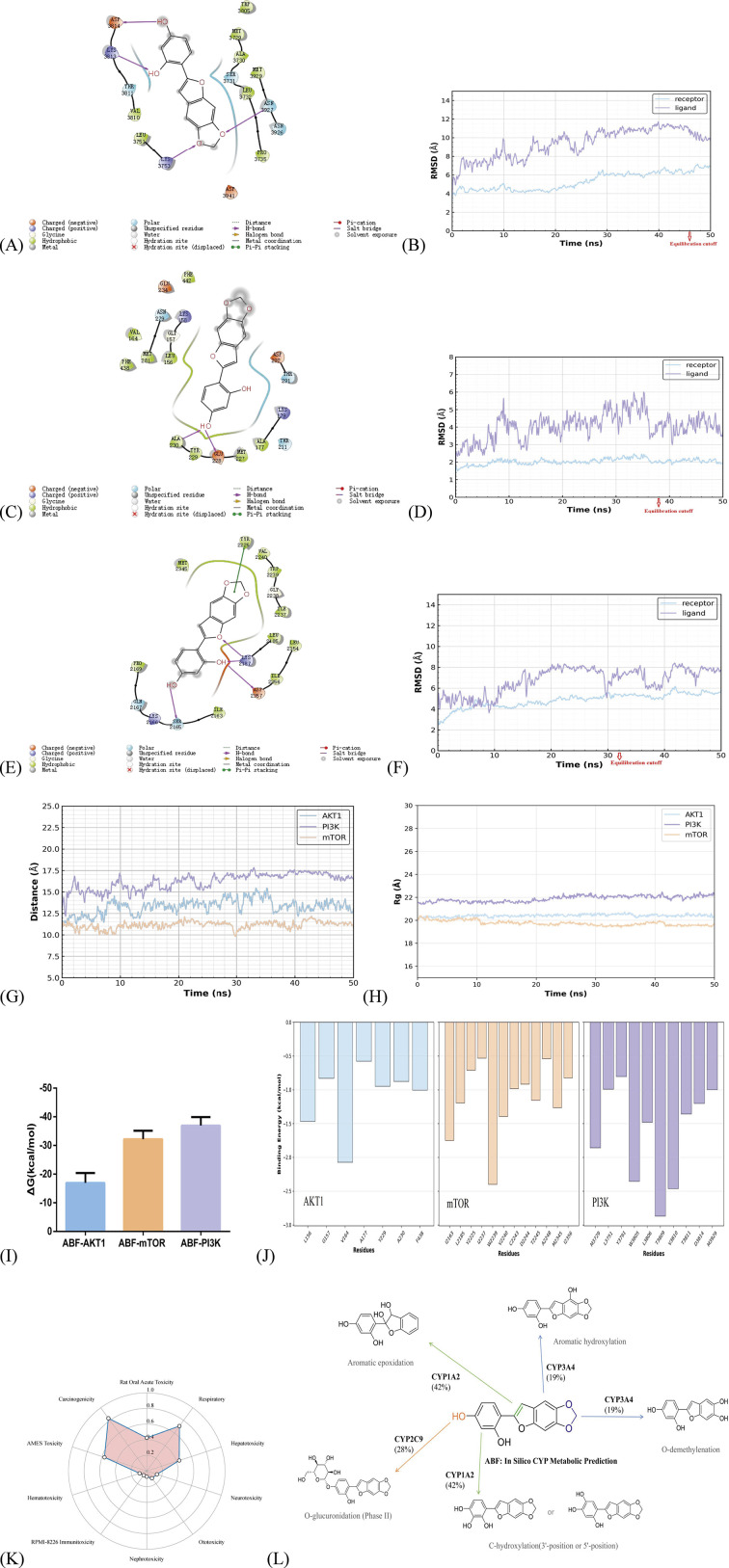
Molecular Dynamics Simulation of ABF and Target Proteins, CYP Enzyme Metabolism Prediction, and Toxicity Prediction. **(A,B)** The ABF-PI3K docking model and RMSD time evolution of the receptor (PI3K) and ligand (ABF) during 50 ns MD simulation. **(C,D)** Docking model of ABF with AKT1 and corresponding RMSD time evolution curve. **(E,F)** The ABF-mTOR docking model and its RMSD. **(G)** Time-dependent distance between ABF and key amino acid residues in the active pockets of AKT1, PI3K, and mTOR. **(H)** Rg profiles of ABF-target complexes. **(I)** Binding free energies (ΔG) of ABF-target complexes. All negative ΔG values indicate favorable binding interactions. **(J)** Per-residue binding energy decomposition of residues contributing favorable binding energy (ΔG < −0.5 kcal/mol) in the ABF-target complexes. **(K)** Predicted toxicity classification of ABF, represented as the probability of being toxic (ranging from 0 to 1). **(L)** The binding probabilities for the main CYP isoforms were as follows: CYP3A4: 42%, CYP1A2: 28%, CYP2C9: 19%.

In the ABF–AKT1 complex (Figure 5C), hydrophobic residues A177, L156, and V164 form a stable hydrophobic pocket around the benzodioxole moiety of ABF. Hydrogen-bonding interactions between the phenolic hydroxyl groups of ABF and A230, Y229, and E228 further anchor the ligand in the ATP-binding pocket and stabilize the complex conformation. For ABF-AKT1 (Figures 5D,G,H), the receptor backbone stabilized at 10 ns (RMSD: 2.0–2.5 Å; Rg: 20.0–20.8 Å), and the ligand conformation converged at 38 ns (ligand RMSD: 3.five to five Å; distance: 12–15 Å).

In the ABF–mTOR complex (Figure 5E), W2239 is the major contributing residue (Figure 5J), while hydrophobic residues including I2163, L2185, and I2356 collectively enclose the aromatic rings of ABF and provide hydrophobic interactions. Hydroxyl groups of ABF form hydrogen bonds with S2165, K2187, and D2357, and π–π stacking with Y2225 is also observed, jointly enhancing the binding affinity. For ABF-mTOR (Figures 5E,G,H): the receptor backbone stabilized at 10 ns (RMSD: 4–6 Å; Rg: 19.3–20.0 Å), and the ligand conformation converged at 32 ns (ligand RMSD: 6–8 Å; distance: 10–12 Å).

In the ABF–PI3K complex (Figure 5A), V3810, L3806, and M3729 wrap around the aromatic core of the ligand. The hydroxyl and ether oxygen atoms of ABF form key hydrogen bonds with D3814, K3813, K3753, and N3927, which are crucial for maintaining ligand orientation and binding stability. For ABF-PI3K (Figures 5A,G,H): The receptor backbone stabilized at 30 ns (RMSD: 6–7 Å; Rg: 21.5–22.5 Å), and the ligand conformation converged at 48 ns (ligand RMSD: 10–11 Å; distance: 16–18 Å).

In short, these conformational diagrams show that the ligand forms a core interaction network through hydrogen bonds and π-π stacking, with hydrophobic interactions further embedding it within the hydrophobic pocket. The synergistic effect of multiple forces ensures stable binding within the pocket. Although fluctuations in the ligand RMSD were observed, they generally trended toward dynamic equilibrium, indicating that the ligand did not dissociate from the binding pocket. Distances between amino acids in the active pocket and the ligand further confirm stable binding within the active site. Over the 50 ns simulation, the Rg values of PI3K, AKT1, and mTOR were maintained stably at around 22 Å, 20.5 Å, and 19.5–20 Å, respectively, with negligible fluctuations. No significant unfolding or conformational collapse was detected, indicating that ABF binding did not impair the global structural integrity of the three kinases and that all systems achieved stable dynamic equilibrium.

Next, average binding free energies (ΔG) were calculated from multiple frames of the MD trajectories: ABF-PI3K complex, −36.92 ± 2.96 kcal/mol; ABF-AKT1 complex, −16.93 ± 3.47 kcal/mol; and ABF-mTOR complex, −32.20 ± 2.67 kcal/mol (Figure 5H). The ΔG values for all three ABF-target complexes were below −9 kcal/mol. According to established criteria, scores below −9.0 kcal/mol indicate strong binding affinity (Friesner et al., 2004). These data confirm that ABF forms stable complexes with the three target proteins, consistent with the strong binding affinities observed in the simulations, theoretically support that ABF can bind to targets in the PI3K/Akt/mTOR, thereby affecting the growth of NPC cells.

Additionally, toxicity prediction of ABF indicates a low probability of hepatotoxicity, nephrotoxicity, and neurotoxicity (Figure 5I). High-score metabolic sites (site score >0.7) were identified: The core phase I metabolic reactions included O-demethylenation of the 5,6-methylenedioxy group, epoxidation of the 2,3-double bond in the benzofuran ring, and aromatic hydroxylation. The main phase II metabolic reaction was glucuronidation at the C4′-position. These predictions can be used to guide subsequent *in vitro* metabolic experiments.

### 3.6 ABF inhibits the PI3K/Akt/mTOR signaling to suppress NPC cell growth

**FIGURE 6:**
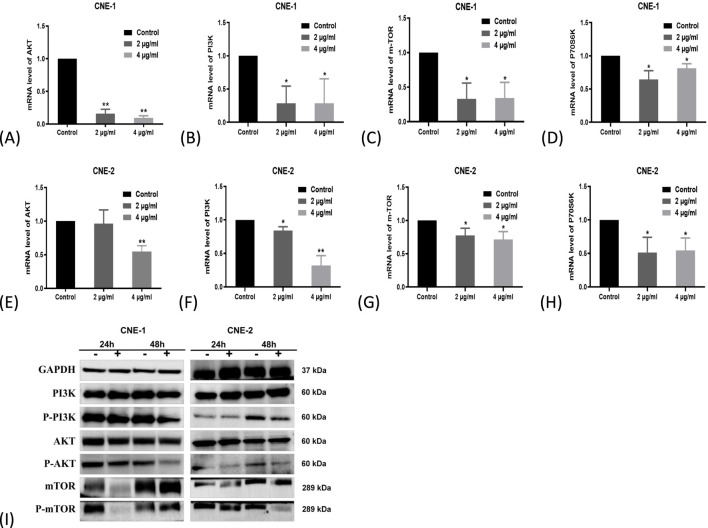
ABF inhibits the growth of NPC cells by regulating the PI3K/AKT/mTOR pathway. **(A–H)** The mRNA expression levels of PI3K, AKT, and mTOR were reduced in the ABF-24h treated groups. **(I)** The levels of the corresponding phosphorylated proteins decreased in the ABF (+) group. Mean ± SD, n = 3/group, Student’s t-test, *p < 0.05, **p < 0.01 vs. control group.

To verify the mechanism by which ABF inhibits NPC growth through this pathway, *in vitro* experiments were conducted to analyze expression changes of molecules in this pathway at the protein and mRNA levels. PCR data showed that following ABF treatment (2, 4 mg/mL) for 24 h, the mRNA levels of PI3K, AKT, m-TOR, and P-70S6K decreased in the 2 cell lines (Figures 6A–H, *p < 0.05). WB results indicated that, compared with the control group, the protein levels of PI3K and AKT in the 4 mg/mL treatment group did not change significantly, while the m-TOR protein level decreased. Moreover, the levels of their phosphorylated forms, p-PI3K, p-AKT, and p-mTOR, all exhibited a decline (Figure 6I). The decrease in protein phosphorylation levels indicates inhibition of protein activity. The trend in mRNA expression levels was consistent with that observed at the protein level. Collectively, These results indicate that the inhibitory effect of ABF on NPC cell growth is closely associated with the downregulation of the PI3K/Akt/mTOR pathway.

### 3.7 ABF suppresses NPC tumor growth *in vivo*

**FIGURE 7:**
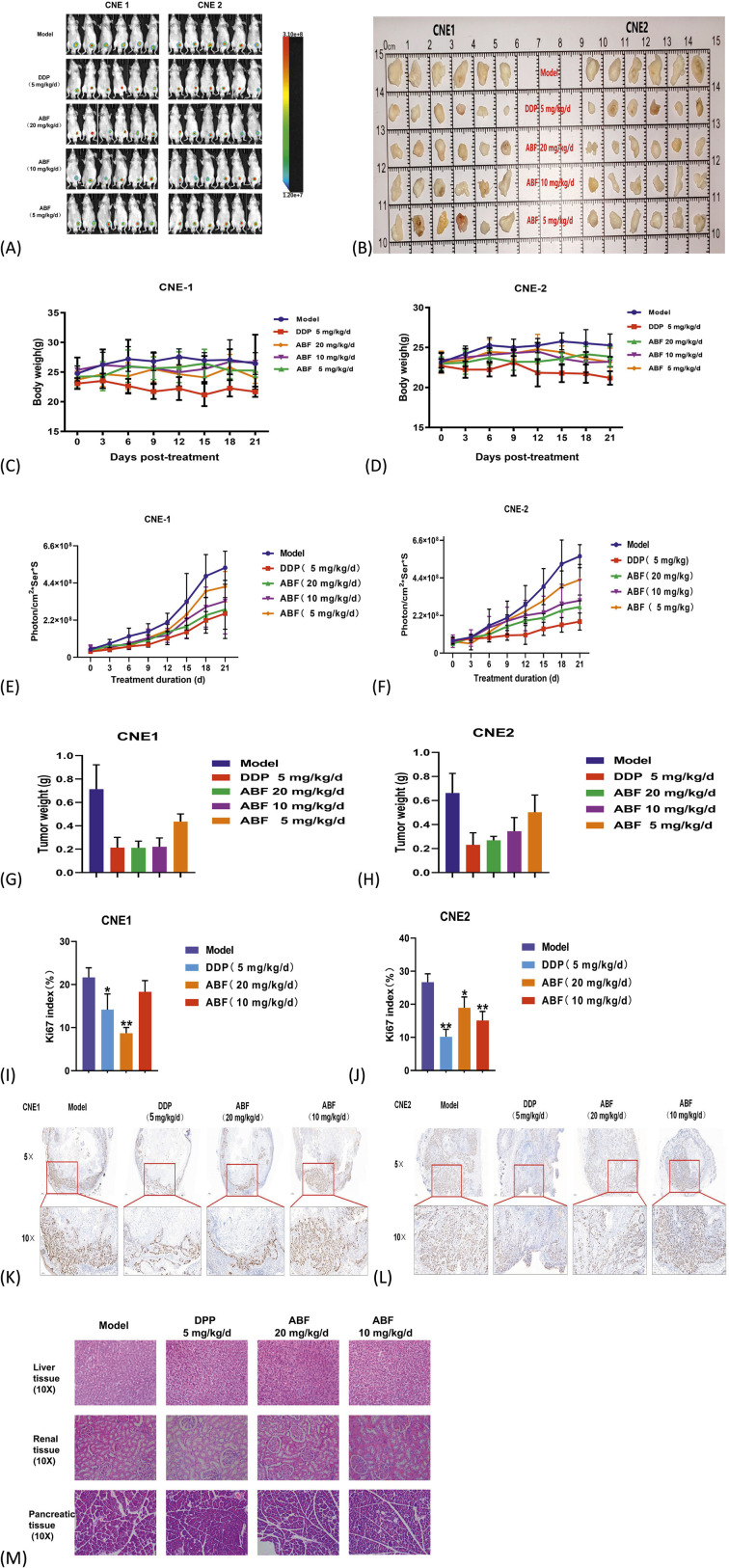
*In Vivo* Anti-Tumor Effect of ABF on NPC. **(A)** Bioluminescence imaging (BLI) of nude mice from the control and ABF-treated groups on 21 days post-treatment,n = 6/group. **(B)** Images of resected xenograft tumor samples of nude mice. **(C,D)** Body weight curve of mice in each group. **(E,F)** Quantitative analysis of BLI signals. **(G,H)** Tumor weight in each group. **(I,J)** Quantitative analysis of Ki67 index. **(K,L)** Ki67 staining results in tumor tissue sections, scale bar,200 μm (above),100 μm (below). **(M)** Representative H&E staining of liver, kidney, and pancreatic tissues from nude mice in each group after treatment. Scale bar, 50 μm. Mean ± SD, n = 6/group, Student’s t-test, *p < 0.05, **p < 0.01 vs. model group.

Subsequently, *in vivo* experiments were performed using a nude mouse model of NPC xenograft tumors to evaluate drug efficacy. Smaller tumors and a significantly slower growth rate were observed in the ABF treatment group compared with the model group (Figures 7A,B). On day 21, the tumor inhibition rates in the high-dose group were 46.59% and 45.71%, respectively (Figures 7E–H). Furthermore, no significant changes in body weight (Figures 7C,D) or histological damage to the liver, kidney, or pancreas (Figure 7M) were observed in the ABF-treated group relative to the model group. The Ki67 Labeling Index serves as the established benchmark for assessing the proliferative activity of tumor cells. A significant decrease in the Ki67 index was observed in the high-dose ABF group relative to the models (Figures 7I,J, *p < 0.05*)*. Immunohistochemistry data revealed that Ki67 positive signals were mainly located in the nuclei of cells at the invasive front of the tumors, presenting as homogeneous, granular, or dot-like brown staining (Figures 7K,L). These results suggested that ABF effectively inhibited the growth of NPC cells in a nude mouse xenograft model.

## 4 Discussion

13 compounds were isolated from the active anti-NPC fraction (chloroform extract) of SDG, and the benzofuran compound ABF was determined to be the most potent anti-NPC monomer among these compounds through activity screening. In terms of potency, ABF exhibited nearly 10-fold lower IC_50_ values against CNE1 and CNE2 cells at 48 h compared to the total extract of SDG. Furthermore, at its IC_50_ concentration, normal nasopharyngeal epithelial NP69 cells still maintained high viability. ABF’s high potency and defined chemical structure make it more amenable to preclinical safety evaluation (e.g., toxicity, metabolism studies) compared with the complex extract, laying the foundation for its subsequent development as a “natural lead molecule”. Previously, only the pharmacokinetic properties of ABF had been reported (Jang et al., 2015), with no mention of its antitumor activity. This study is the first to confirm the anti-tumor activity of ABF against NPC cells, and provides pharmacological evidence to elucidate the traditional applications of SDG.

The high metastatic propensity of NPC is a major cause of poor patient prognosis, and EMT is a core pathological process enabling tumor cell migration and invasion (Pastushenko and Blanpain, 2019). Wound healing and transwell assays revealed the mechanism underlying the anti-metastatic effect of ABF against NPC cells: by inhibiting the EMT process, ABF blocks the mesenchymal transition of cells, leading to reduced cell adhesion and weakened motility, as evidenced by decreased migration distance and fewer invading cells. Cell cycle analysis showed that S-phase arrest may block or disrupt DNA replication, thereby delaying cell proliferation. Furthermore, apoptosis data confirmed that ABF induces NPC cell death via the intrinsic (mitochondrial) apoptotic pathway: a decreased Bcl-2/Bax ratio disrupts mitochondrial membrane potential, triggering cytochrome c release, which in turn activates caspase-9. Caspase-9 then cleaves and activates downstream effectors caspase-3 and caspase-7, which degrade PARP (a DNA repair enzyme). This cleavage not only serves as a hallmark of apoptosis but also accelerates programmed cell death by impairing DNA repair capacity (Liu et al., 2020; Riedl and Shi, 2024).

The PI3K/Akt/mTOR pathway is a key signaling pathway aberrantly activated in NPC (Li et al., 2020). *In vitro* experiments observed that PI3K/AKT/mTOR dephosphorylation occurred within 24 h, well before the onset of apoptosis and cell cycle perturbations, supports the notion that pathway inhibition is an early effect of ABF. As a central upstream hub linking cell proliferation, apoptosis, and EMT (Chen et al., 2025), we speculate that this pathway is involved in the antitumor effects of ABF. Although the data suggest that ABF treatment is associated with inhibition of the PI3K/AKT/mTOR pathway, the current evidence supports a correlative rather than a definitive causal relationship. Future studies utilizing functional rescue experiments are warranted to establish direct causality. It should be noted that, given the involvement of the PI3K/AKT/mTOR pathway in numerous homeostatic functions, the possibility of off-target effects cannot be overlooked. Current experiments do not fully exclude impacts on other signaling nodes, and further studies (e.g., kinase profiling or genetic knockout) are needed to confirm the compound’s direct target.

From a structure-activity relationship perspective, the substitution at the C-2 and C-3 positions of the benzofuran core ring, along with various substituents on the benzene ring, encompasses most benzofuran-derived ring systems. It is this unique heterocyclic core structure and the diversity of substituents that endow benzofuran compounds with a wide range of biological activities and application value (Farhat et al., 2020; Patel et al., 2024). We believe that the anti-NPC activity of ABF is closely related to its unique substituent distribution: preliminary studies indicate that the substituent at the C-2 position of the benzofuran ring is a key site for the compound’s cytotoxic activity, and different substituents on the heterocycle at the C-2 position also have a certain impact on cytotoxicity (Hayakawa et al., 2003). The C-2 substituent of ABF is a 2′,4′-dihydroxyphenyl group. The hydrogen bonding interactions are primarily provided by the two hydroxyl groups on the 2′,4′-dihydroxyphenyl moiety, which serve as the main force stabilizing ABF within the binding pocket (Figure 5). Previous studies have shown that benzofuran skeletons containing a 2′,4′-dihydroxyphenyl group possess good anticancer potential (Czerwonka et al., 2018; Katsanou et al., 2007). The methylenedioxy group in drug design is primarily associated with improving pharmacokinetic properties, often used to optimize metabolic stability, lipophilicity, and permeability, as well as to modulate the efficiency of drug-target binding (Branches et al., 2024; Li, 2020). Studies have shown that in benzofuran systems, anticancer potential is directly related to lipophilicity (Carella et al., 2019). Therefore, the 5,6-methylenedioxy ring in ABF may enhance the molecule’s lipophilicity, facilitating penetration through tumor cell membranes, which is crucial for entering cancer cells to exert effects. Additionally, the conjugated system of the benzofuran ring itself may help stabilize free radicals or participate in electron transfer processes (Galal et al., 2009), which could play a role in anticancer mechanisms. It has been reported that the conjugated planar structure of the benzofuran ring may interfere with cancer cell proliferation-related pathways by binding to hydrophobic pockets of target proteins (Gao et al., 2020; Ghosh et al., 2020; Kanamori et al., 2014). Molecular docking (light blue arcs: hydrophobic interactions, Figure 5) also illustrates that the aromatic and oxygen-containing heterocyclic moieties of ABF are surrounded by hydrophobic residues of the protein, forming a hydrophobic pocket that firmly anchors the ligand through hydrophobic effects. In summary, based on the molecular docking modeling and literature precedence, we speculate that the anti-nasopharyngeal carcinoma activity of ABF may be closely related to the synergistic effects of the rigid conjugated system of the benzofuran core, the methylenedioxy ring optimizing pharmacokinetic properties, and the 2′,4′-dihydroxyphenyl functional group in its molecular structure. Of course, future structure–activity relationship (SAR) studies are necessary to ultimately validate this hypothesis.

Notably, we hypothesize that the anti-NPC effect of ABF may be achieved through the inhibition of multiple PI3K isoforms rather than targeting a single isoform. The structural characteristics of ABF suggest that it tends to act as a pan-inhibitor or possesses weak isoform bias, lacking specific selectivity. First, ABF lacks the side chains (e.g., piperazine, pyrimidine rings) present in selective inhibitors (such as alpelisib and idelalisib) that enable precise interaction with non-conserved residues of specific isoforms (Perreault et al., 2020). The 2′,4′-dihydroxyphenyl substituent on its core structure aligns with the definition of benzofuran-type polyphenolic compounds, making it more similar to natural polyphenolic pan-PI3K inhibitors like quercetin (Boo et al., 2025). Thus, ABF is more likely to follow this trend rather than demonstrate strong isoform selectivity. Second, ABF has a moderate molecular weight that allows it to enter the ATP-binding pockets of all Class I PI3K isoforms, yet it lacks the bulky or charged groups required to distinguish differences among isoform pockets, making it difficult to achieve highly specific binding to any single isoform (Miller et al., 2019). Molecular docking results (Figure 5A) show that hydrophobic residues envelop the aromatic core of the ligand, while the phenolic hydroxyl groups form hydrogen bonds with residues such as Val and Met in the hinge region of PI3Kγ, consistent with the typical binding mode for γ/δ isoformss (Ma et al., 2018; Miller et al., 2019). Hydrophobic interactions are critical for inhibitor binding, and the hydrophobic benzofuran scaffold of ABF may predispose it to bind to the hydrophobic pockets of PI3Kγ/δ (which are more hydrophobic in these two isoforms), resulting in a slight preference for the γ/δ isoforms. However, this preference is far from the level of selective inhibitors. Admittedly, these insights are based on structural predictions and require further validation through isoform-specific enzymatic assays.

As a natural potential anti-NPC agent, ABF exhibited IC_50_ values of approximately 16.78 μM and 14.56 μM (after conversion) in CNE1 and CNE2 cells, respectively. Although its *in vitro* activity is lower than that of rapamycin analogs (e.g., sirolimus, RAD001) and the dual PI3K/mTOR inhibitor BEZ235, both of which show IC_50_ values in the nm range, it is comparable to that of the first-generation PI3K inhibitor LY294002, whose IC_50_ is approximately 10–20 μM (Occhiuzzi et al., 2023; Sabbah et al., 2024). Despite its relatively high *in vitro* IC_50_, ABF is of natural origin and possesses favorable biocompatibility. Our group previously reported its reversal effect on drug-resistant CNE-1/DDP and CNE-2/DDP cells (He, 2022), suggesting an advantage of low propensity for drug resistance, which may provide new strategies for addressing NPC chemoresistance. LY294002 shows stable inhibitory efficacy but has been discontinued from clinical development due to unfavorable pharmacokinetics and dose-limiting toxicity; it is now widely used only as a research tool. ABF achieved tumor growth inhibition rates of 46.59% and 45.71% in two cell-derived xenograft models, respectively, with no obvious body weight loss or histological toxicity. However, systematic toxicological evaluations are still lacking, including measurements of serum biochemical markers (ALT, AST, ALP, CRE, BUN) and comprehensive histopathological analysis. Future studies will aim to establish a complete safety evaluation profile for ABF, including comparative analyses with LY294002, to fully characterize its safety properties. In addition, detailed pharmacokinetic parameters of ABF in mice, such as plasma concentration, half-life, and tissue distribution, have not yet been determined. Although the observed *in vivo* efficacy is encouraging, further pharmacokinetic/pharmacodynamic studies are required to clarify the exposure–response relationship and optimize the dosing regimen, providing a scientific basis for translational research.

This study identified the benzofuran monomer ABF from the medicinal herb SDG through HPLC fingerprinting and multi-step chromatographic separation and identification. The results confirmed that the *in vitro* anti-NPC activity of ABF is significantly superior to that of the SDG extract, establishing ABF as the key active component of SDG against NPC. Through *in vitro* and *in vivo* activity assays, the anti-NPC efficacy of ABF was verified, and its mechanism of action and druggability potential were preliminarily elucidated. Although this study confirms the anti-NPC activity of ABF *in vitro* and *in vivo*, further investigations are required to evaluate its feasibility as a molecule candidate. Future research will focus on extending the evaluation to a broader panel of NPC cell lines, as well as conducting pharmacokinetic and systematic toxicology assessments, to steadily promote the translational development of ABF.

## Data Availability

The original contributions presented in the study are included in the article/Supplementary Material, further inquiries can be directed to the corresponding authors.
