# Supplementary material for: Benzofuran compound from Sophora tonkinensis Gagnep suppresses nasopharyngeal carcinoma growth via PI3K/AKT/mTOR pathway: evidence from molecular dynamics simulation and in vitro experiments
Source: Front Pharmacol. 2026 Apr 23;17:1778697. doi: 10.3389/fphar.2026.1778697 (PMC13149249; doi:10.3389/fphar.2026.1778697)
Supplement: Supplementary file 1 [file DataSheet1.pdf]

Supplementary materials

For

(2',4'-Dihydroxyphenyl)-5,6-methylenedioxybenzofuran from Sophora tonkinensis Gagnep. Suppresses Nasopharyngeal Carcinoma Growth via PI3K/AKT/mTOR pathway: evidence from molecular dynamics simulation and in vitro experiments

Peiying Wu[a,1],Xiaomei Gong[a,1], Wenyu Zhang[b], Dandan Mo[c], Lili He[a], Zhijun Song[a], Chunli Ou[c], Meng Li[a], Xiaolei Zhou[c], Xingyu Xiao[a], Xianghua Xia[a], *,ShuoWang[a, **]

a National Engineering Research Center of Southwest Endangered Medicinal Resources Development, Guangxi Botanical Garden of Medicinal Plants, Nanning, China

b Guangxi Medical University Cancer Hospital,Nanning, China

c Guangxi Key Laboratory of Medicinal Resources Protection and Genetic Improvement, Guangxi Botanical Garden of Medicinal Plants, Nanning, China

*** Correspondence:**

Xianghua Xia*, ShuoWang*

wangshuo@gxyyzwy.com(S. Wang)

Keywords: nasopharyngeal carcinoma; sophora tonkinensis gagnep; benzofuran compound; Apoptosis; PI3K/AKT/ mTOR/signaling pathway;

Contents

1 Traditional Uses and Distribution of Sophora tonkinensis Gagnep 1

2 Western blotting and RT-PCR experiments detail 3

3 Protein raw data 7

1 Traditional Uses and Distribution of Sophora tonkinensis Gagnep

Table S1.Traditional Uses and Distribution of Sophora tonkinensis Gagnep

Local Name	Ethnic Medicinal Use + Part Used	Pharmacological Application + Part Used	Country/Place of Origin	References
Shan Dou Gen	Traditional Chinese Medicine: Relieving sore throat and swollen gums.(Root and rhizome)	Anti-inflammatory, antiviral, immunomodulatory; Root and rhizome	Southwest China	1.Pharmacopoeia Commission of the People's Republic of China. Pharmacopoeia of the People's Republic of China (Volume 1)[S]. Beijing: China Medical Science Press, 2020: 28<br>2.Zeng F-F, Chen Z-H, Luo F-H, Liu C-J, Yang X, Zhang F-X, et al. Sophorae tonkinensis radix et rhizoma: A comprehensive review of the ethnopharmacology, phytochemistry, pharmacology, pharmacokinetics, toxicology and detoxification strategy. J. Ethnopharmacol.2025; 337: 118784. https://doi.org/10.1016/j.jep.2024.118784
Rang Du Ba, Guang Dou Gen	Zhuang Medicine: Relieving laryngeal wind, laryngeal abscess and laryngitis. (Root and rhizome)	Antitumor, anti-inflammatory; Root and rhizome	Guangxi, China	3.Guangxi Zhuang Autonomous Region Administration of Traditional Chinese Medicine. Records of Ethnic Medicines in Guangxi (Volume 1). Nanning: Guangxi Science and Technology Press, 2004.
Shan Dou Gen	Yao Medicine: Relieving pharyngitis and swollen gums. (Root and rhizome)	Anti-inflammatory, analgesic; Root and rhizome	Yao inhabited areas of Yunnan and Guangxi, China	4.Li Z, Wei JY. Yao Pharmacology. Guangxi Science and Technology Press.Nanning,2003: 131-132.
sanb dub get	Hmong Medicine: Treating cough, sore	Anti-inflammatory, antibacterial, antiviral; Root and	Guizhou, China	5.Tang HH. Hmong Pharmacology. Guiyang: Guizhou Ethnic Publishing House, 2011: 107

	throat and jaundice. (Root and rhizome)	rhizome		6.Tian HY, Du J. Hmong Medicine and Pharmacology. Guiyang: Guizhou Ethnic Publishing House, 1992: 226.
Shan Dou Gen	Dong Medicine: Relieving sore throat and pyogenic skin infections. (Root and rhizome)	Anti-inflammatory, antibacterial; Root and rhizome	Dong inhabited areas of Guizhou and Guangxi, China	7.Wang Y, Tian L, Tian HY. History of Dong Medicine in China. Beijing: Ancient Books Press of Traditional Chinese Medicine, 2013: 89-90.
Shan Dou Gen	Buyi Medicine: Clearing away heat and toxic materials, reducing swelling and alleviating pain; treating sore throat and abscesses.(Root and rhizome)	Anti-inflammatory, analgesic; Root and rhizome	Buyi inhabited areas of Guizhou, China	8.Pan LT, Zhao JH, Zhang JM. Buyi Medicine. Guiyang: Guizhou Science and Technology Press, 2003: 156.
Sữa Đậu Núi、Rễ Đậu Núi (Vietnamese)	Vietnamese Traditional Medicine: Treating tonsillitis, pharyngitis, cough, sore throat, jaundice and swollen gums; external use for snake bites and burns. (Root and rhizome)	Anti-inflammatory, antiviral, antitumor; Root and rhizome	Northern Vietnam	9.Nguyen Van Duong. Medicinal Plants of Vietnam. Hanoi: Hanoi Publishing House, 1993: 245.<br><br>10.https://thuocdantoc.vn/duoc-lieu/son-dau-can

2 Western blotting and RT-PCR experiments detail

2.1 Western Blot (WB) Assay

Total Cellular Protein Extraction
After cells were treated with drugs for 24, 48 and 72 h, they were rinsed twice with 1 × PBS and lysed with a lysis buffer prepared by mixing buffer solution, protease inhibitor and PMSF at a volume ratio of 100:1:1:1. Lysis was performed on ice for approximately 15 min, followed by transferring the lysate to an EP tube and centrifuging at 12,000 rpm for 15 min at 4 °C. The supernatant was collected for subsequent experiments.
Determination of Total Cellular Protein Concentration (BCA Method)
A BCA Protein Quantification Kit was used to measure protein concentration, with a standard curve prepared in accordance with the kit instructions. The working solution was freshly prepared by mixing Solution A and Solution B from the BCA kit at a volume ratio of 50:1, with 200 µl of the working solution added to each well. In a 96-well plate, 20 µl of protein standard and 20 µl of the sample protein solution to be tested were added to separate wells, followed by the addition of 200 µl of the pre-prepared BCA working solution to each well. The 96-well plate was wrapped with tin foil to avoid light and incubated at 37 °C for 30 min. The absorbance value was measured at a wavelength of 562 nm using a microplate reader.
Electrophoresis and Membrane Transfer
Protein denaturation: The protein samples were heated at 95 °C for 10 min.**Protein electrophoresis**: A 20 µl volume of denatured protein sample was loaded into each well, with 5 µl of pre-stained protein marker added simultaneously. Electrophoresis was initiated at a voltage of 90 V; the voltage was adjusted to 40 V if only one gel was placed in the electrophoresis tank. When the bromophenol blue front reached the separating gel, the voltage was increased to 120 V and electrophoresis continued until the protein marker migrated to an appropriate position, then the electrophoresis was terminated.
Membrane transfer: The PVDF membrane sandwich transfer method was adopted, with constant current set at 150 mA for 80 min.
Blocking: The PVDF membrane was blocked with 5% nonfat milk on a shaker at room temperature for 1.5 h.
Primary antibody incubation: The blocked PVDF membrane was removed, the blocking buffer was discarded, and the membrane was rinsed twice with 1 × TBST for 5 min each time. Primary antibodies were diluted with primary antibody dilution buffer at the specified ratios (Table S1), and the membrane was incubated with the diluted primary antibodies overnight at 4 °C.
Secondary antibody incubation: After recovering the primary antibodies, the membrane was rinsed three times with 1 × TBST. Secondary antibodies were diluted with 1 × TBST at a volume ratio of 1:5000, and the membrane was incubated with the diluted secondary antibodies at room temperature for 1 h, followed by three rinses with TBST after incubation.
Development: The developing solution was freshly prepared by mixing Solution A and Solution B at an equal volume ratio of 1:1 in accordance with the kit instructions, protected from light. The PVDF membrane was incubated with the developing solution for 2 min, then transferred to an imaging system for scanning and imaging. The gray values of the protein bands were analyzed using gel image analysis software.

2.2Quantitative Real-Time Polymerase Chain Reaction (qRT-PCR) Assay

Total cellular RNA extraction: Total cellular RNA was extracted following the instructions of the Total RNA Extraction Kit. After 48 h of drug treatment, cells were rinsed twice with 1 × PBS buffer. 300 µL of RNA lysis buffer and 300 µL of RNA dilution buffer were added to each well for cell lysis for 3 min, and the lysate was transferred to a 1.5 mL RNase-free EP tube. The tube was centrifuged at 14,000 rpm for 8 min in a pre-cooled centrifuge at 4 °C, and the supernatant was collected. The supernatant was mixed with anhydrous ethanol at a volume ratio of 2:1, and the mixture was transferred to a spin column, followed by centrifugation at 14,000 rpm for 1 min; the filtrate was discarded, and the spin column was washed twice with 600 µL of RNA washing buffer, with the filtrate discarded after each centrifugation. 50 µL of DNA incubation mixture

was added to the spin column and incubated at room temperature for 15 min, then 600 μL of RNA washing buffer was added, and the filtrate was discarded by centrifugation. After two additional washes, the spin column was centrifuged for 2 min without adding washing buffer to remove residual liquid. The spin column was then placed into a clean elution tube, 50 μL of RNase-free water was added and allowed to stand for 2 min, followed by centrifugation at 14,000 rpm for 1 min to elute RNA. The extracted total RNA was aliquoted and stored at -80 °C for subsequent use.

Determination of total RNA purity and integrity: A double-beam ultraviolet spectrophotometer was used to detect RNA concentration and purity, and agarose gel electrophoresis was performed to verify RNA integrity. A 1% agarose gel was prepared with 1 × TBE buffer. 1 μL of 6× Loading buffer was mixed with 5 μL of RNA solution, and the mixture was loaded into the sample well of the agarose gel. Electrophoresis was carried out at 80 V for 45 min, and the results were observed and analyzed using a gel imaging system.

Primer design: All primers were synthesized by Sangon Biotech (Shanghai) Co., Ltd., and the primer sequences are listed in Table.S 2.

Reverse transcription: After confirming the concentration and integrity of total RNA, reverse transcription was performed on ice in accordance with the instructions of the 5× All-In-One RT MasterMix Kit, with the reaction systems prepared as follows.

First-strand reaction system (8 μL total volume):
Total RNA: 1 μl
AccuRT Reaction Mix: 2 μL
RNase Free ddH2O: up to 8 μL

The mixture was gently mixed and incubated at room temperature for 5 min, then the following components were added to form the final reverse transcription system (20 μL total volume). The system was placed in a PCR instrument and run with the following program: 25 °C for 10 min, 42 °C for 50 min, and 85 °C for 5 min to reverse-transcribe RNA into cDNA.

Final reverse transcription system (20 μL total volume):
Above reaction mixture: 8 μL
AccuRT Reaction Stopper (5×): 2 μL
5× All-In-One RT MasterMix: 4 μL
RNase Free ddH2O: 6 μL

qRT-PCR detection of PI3K and mTOR mRNA expression (GAPDH as the internal reference): Using the synthesized cDNA as the template, three replicate wells were set up for each sample, and one negative control well was set for each target gene. The qRT-PCR reaction system was prepared as follows, and amplification was performed with 40 cycles of the following program: 94 °C for 30 s (pre-denaturation), followed by 40 cycles of 94 °C for 5 s (denaturation), 60 °C for 15 s (annealing), and 72 °C for 10 s (extension).

qRT-PCR reaction system (20 μL total volume):
cDNA template: 2 μL
Primer Mix (10 μM): 0.8 μL
2× Taq Master Mix: 10 μL
RNase Free ddH2O: 7.2 μL

(6) **Data analysis**: The blank control group was used as the reference to adjust the baseline, and the average Ct value of each gene was calculated. With GAPDH as the internal reference gene, the relative mRNA expression levels of each target gene (PI3K and mTOR) were calculated using the 2-△△CT method.

Table S1. Western blotting antibody information

Antibody	Dilution ratio	Cat number	Company
Bcl-2	1:1000	4223T	Gene Tex
Bax	1:1000	5023T	Gene Tex
Caspase-3	1:1000	9662S	Cell signaling
Cleaved Caspase-3	1:1000	9661T	Cell signaling
Caspase-7	1:1000	9492T	Cell signaling
Cleaved Caspase-7	1:1000	8438T	Cell signaling
Caspase-8	1:1000	4790T	Cell signaling
Cleaved Caspase-8	1:1000	9496T	Cell signaling
Caspase-9	1:1000	9502T	Cell signaling
Cleaved Caspase-9	1:1000	71237S	Cell signaling
PARP	1:1000	9542T	Cell signaling
Cleaved PARP	1:1000	5625T	Cell signaling
AKT	1:1000	9272S	Cell signaling
Phospho-AKT	1:1000	9271T	Cell signaling
Pi3k	1:1000	4257T	Cell signaling
Phospho-Pi3k	1:1000	42287	Cell signaling
m-TOR	1:1000	2983T	Cell signaling
Phospho-m-TOR	1:1000	5536T	Cell signaling
Vimentin	1:1000	5741T	Cell signaling
Snail	1:1000	3879T	Cell signaling
β-Catenin	1:1000	9562S	Cell signaling
E-Cadherin	1:1000	SI95T	Cell signaling
GADPH	1:10000	5174S	Cell signaling
Rabbit LgGanibody (HRP)	1:1000	43052	Gene Tex
Ki67	1:1000	12202T	Cell signaling

Table S2. Primer sequences for real-time quantitative PCR.

Gene	Forward primer (5′-3′)	Reverse primer (5′-3′)
PI3K	5′ -TGGCTTAAAGAATACAACTCTGGG - 3′	5′ -AATAAAAGGCACTCGCTCCC - 3′
AKT	5′ -TCACACCACCTGACCAAGATG- 3′	5′ -GCTGGCCGAGTAGGAGAACT- 3′
mTOR	5′ -TTACCCCCTTCACCAGTTTCC - 3′	5′ -ATCAGCGAGTTCTTGCTATTCC- 3′
P70S6	5′ -TTTTACCCAGCCCCGGA- 3′	5′ -ATCAGCGAGTTCTTGCTATTCC- 3′
GAPDH	5′ -GAGCGAGATCCCTCCAAAAT - 3′	5′ -TGAGTCCTTCCACGATACCAAA - 3′

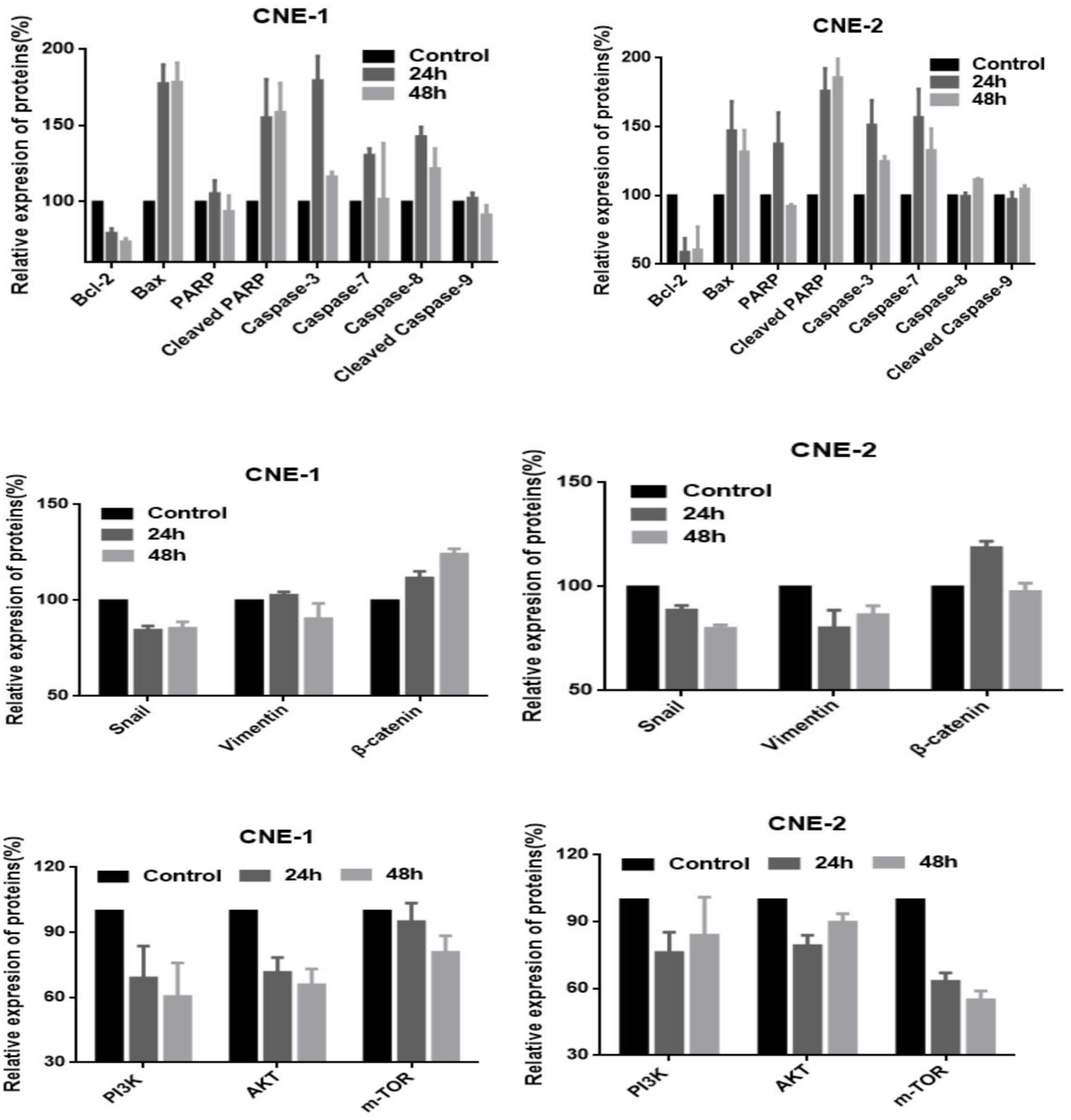

Fig. S1 Western blot analysis of signaling pathway-related protein expression. Semi-quantitative analysis was performed using GAPDH as the internal reference.

3 Protein raw data

CNE1

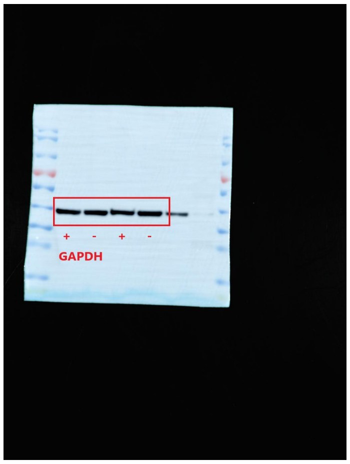

GAPDH

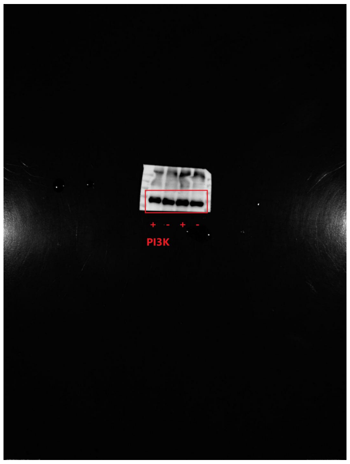

PI3K

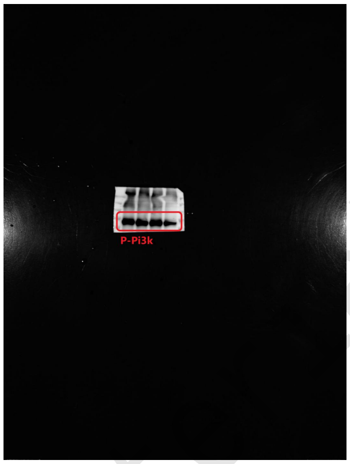

P-PI3K

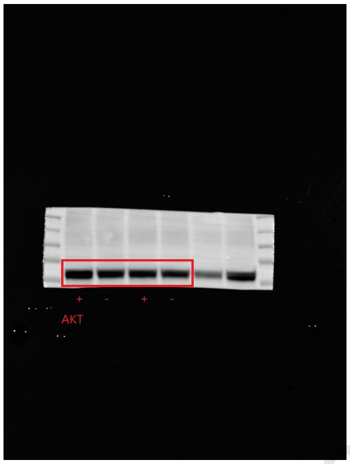

AKT

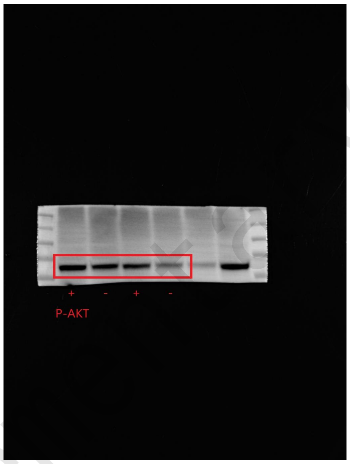

P-AKT

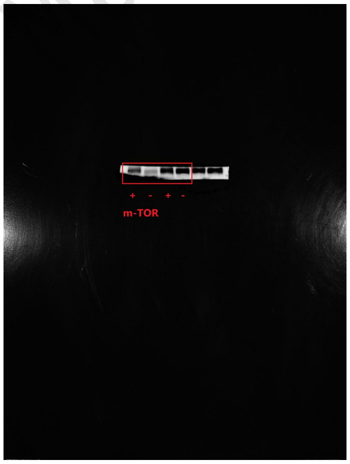

m-TOR

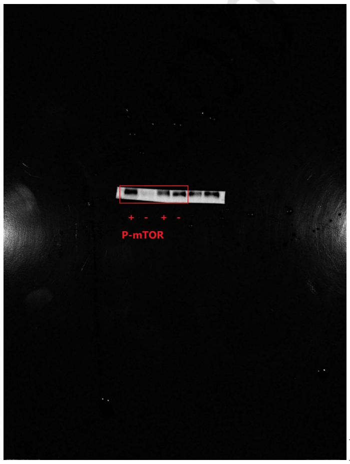

P-m-TOR

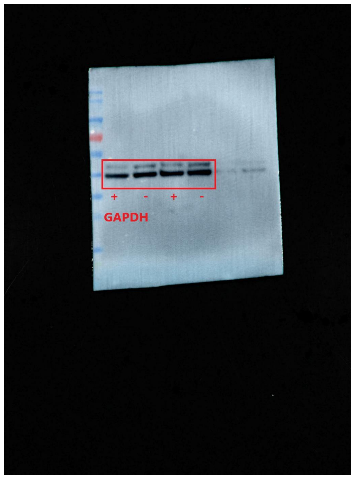

GAPDH

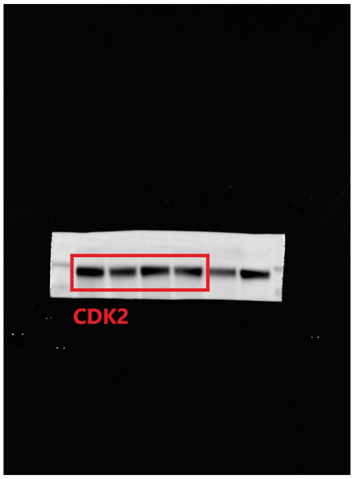

CDK2

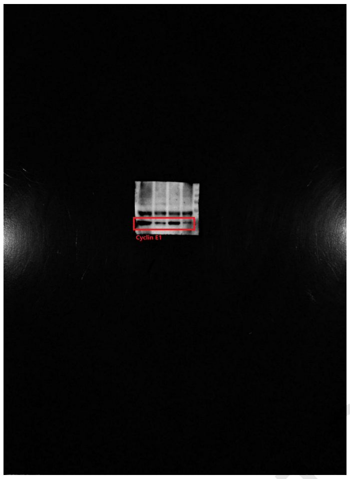

Cyclin E1

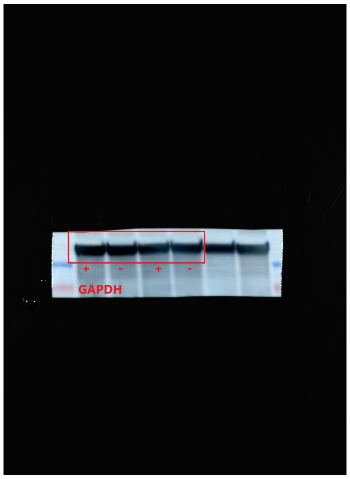

GAPDH

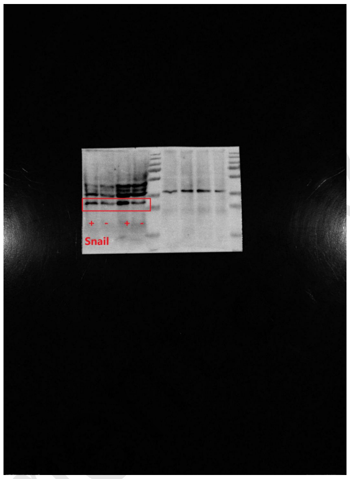

Snail

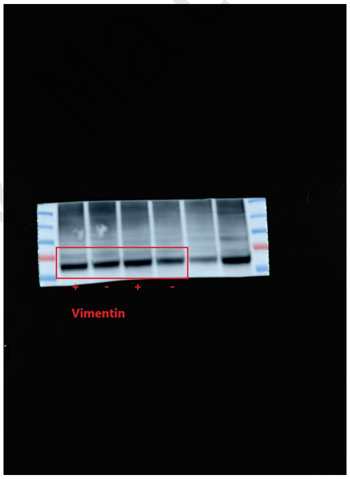

Vimentin

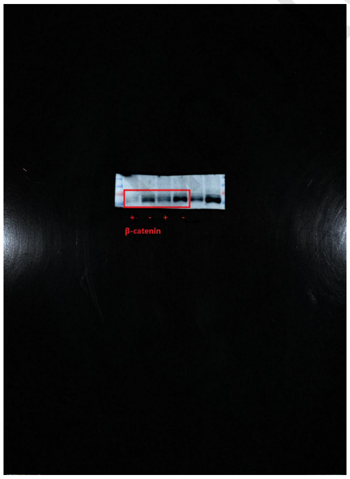

β-catenin

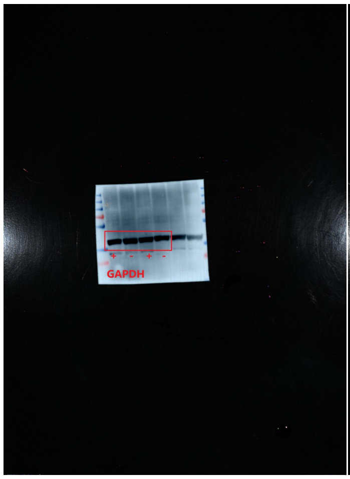

GAPDH

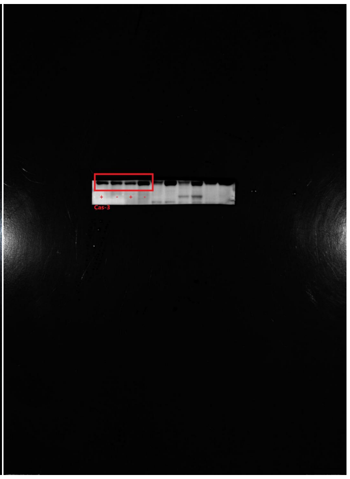

Caspase3

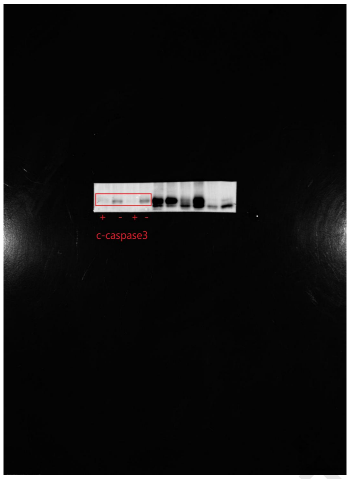

Cleaved Caspase3

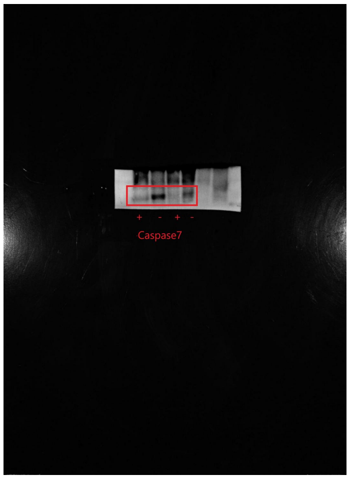

Caspase7

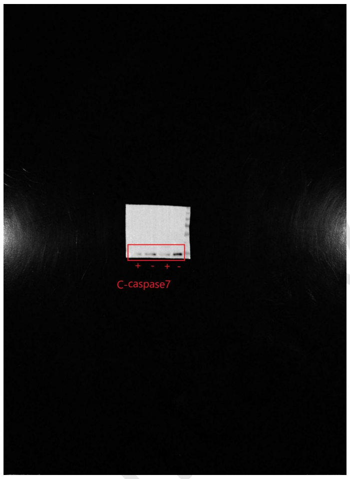

Cleaved Caspase7

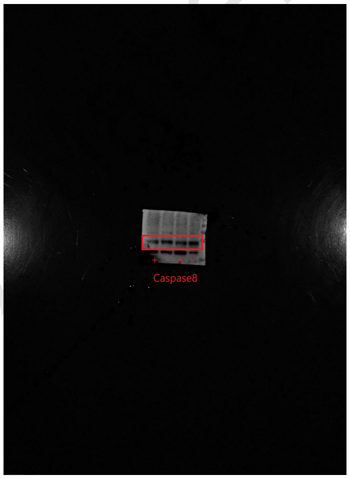

Caspase8

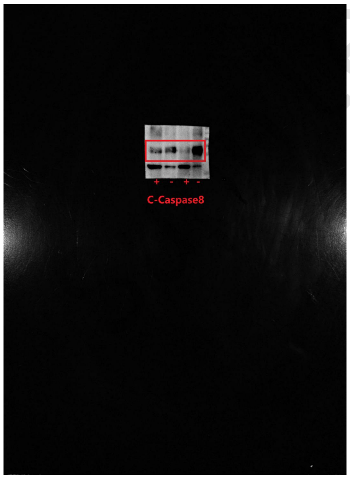

Cleaved Caspase8

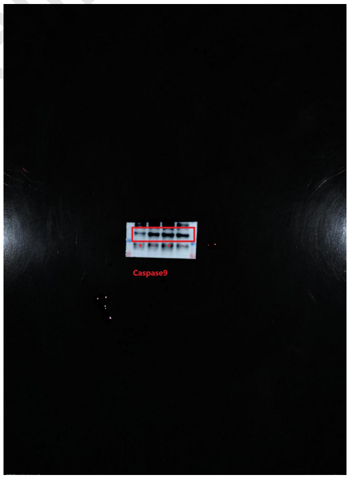

Caspase9

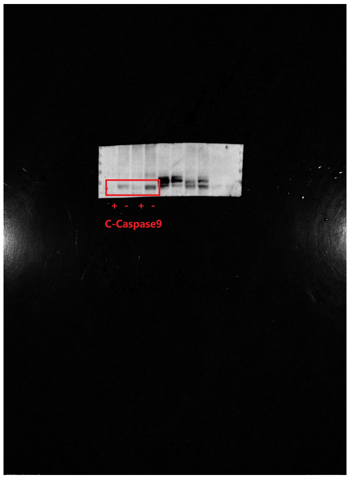

Cleaved Caspase9

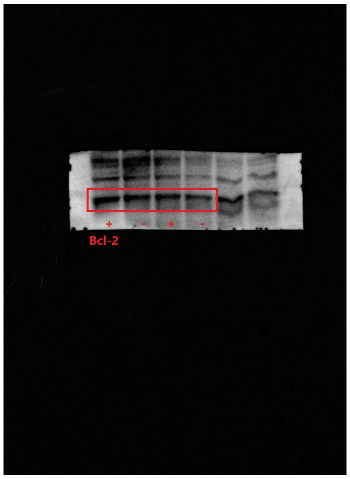

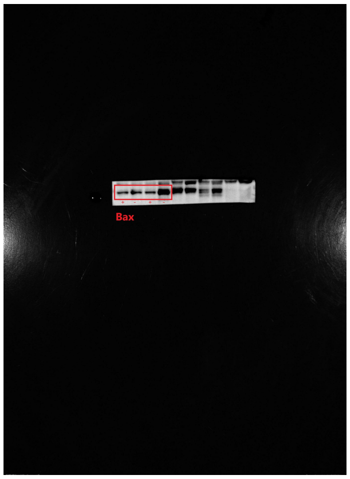

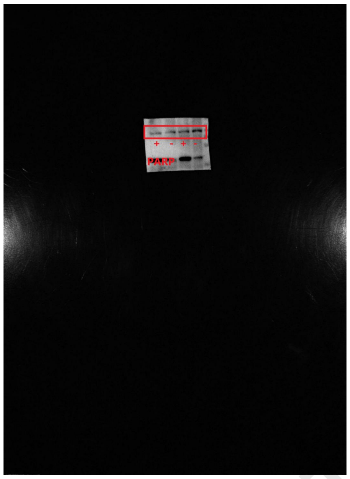

Bcl-2 **BaX** **PARP**

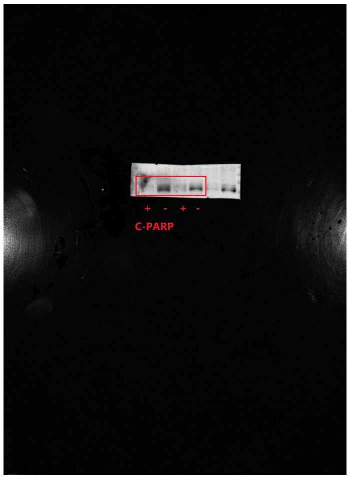

Cleaved PARP

Supplementary Materials

CNE2

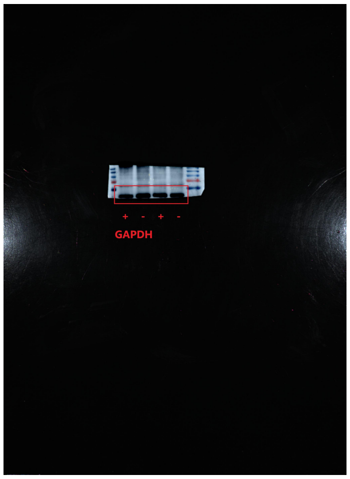

GAPDH

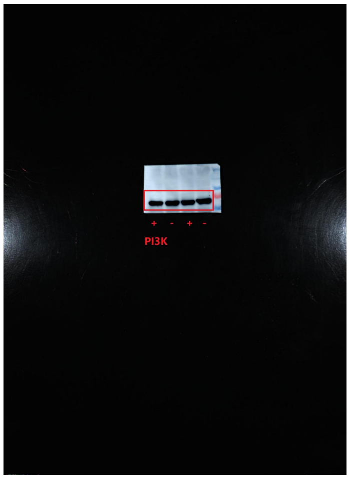

PI3K

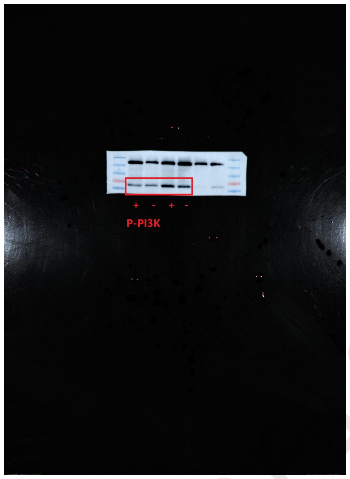

P-PI3K

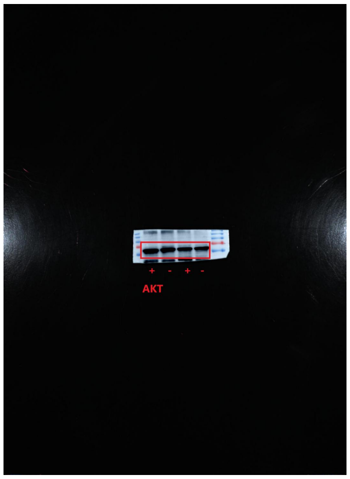

AKT

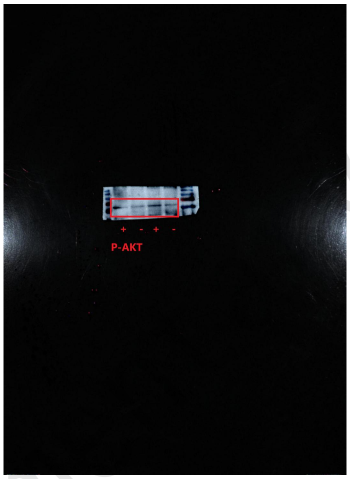

P-AKT

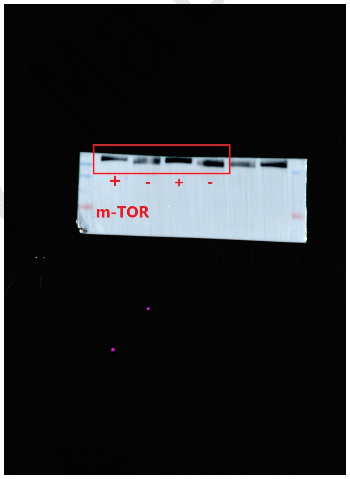

m-TOR

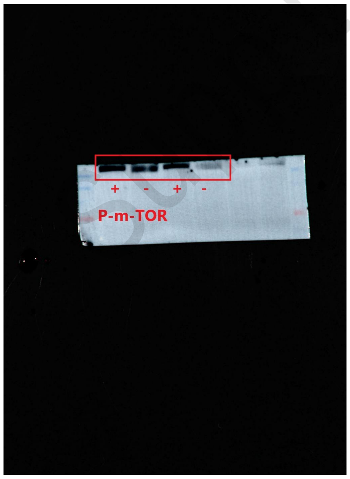

P-m-TOR

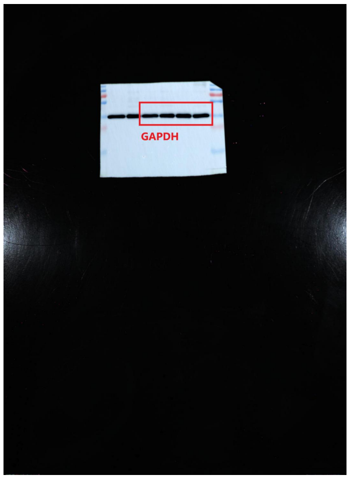

GAPDH

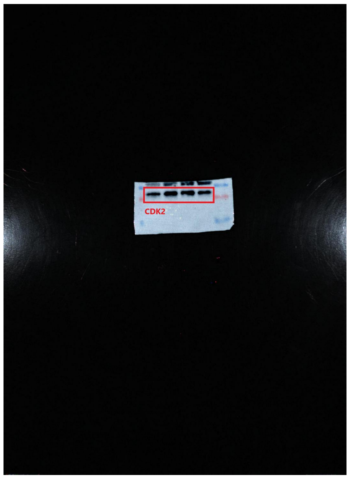

CDK2

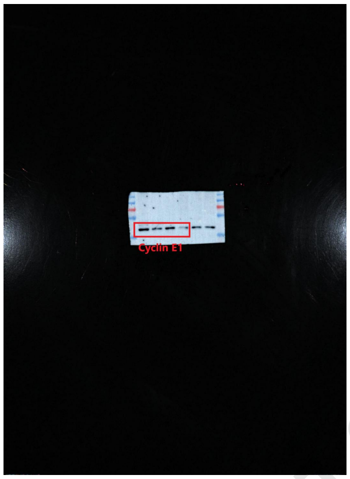

Cyclin E1

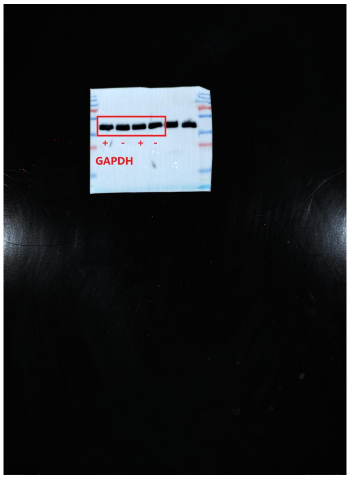

GAPDH

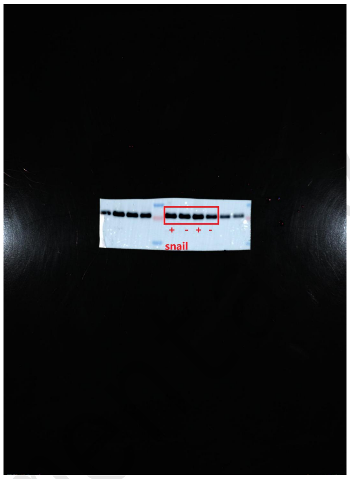

Snail

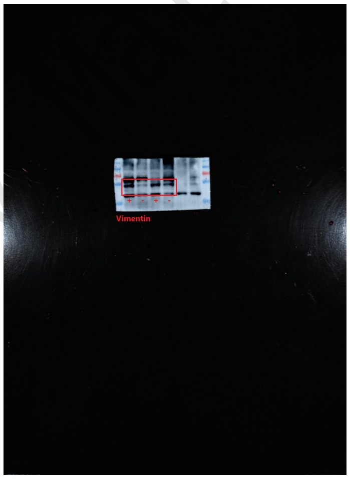

Vimentin

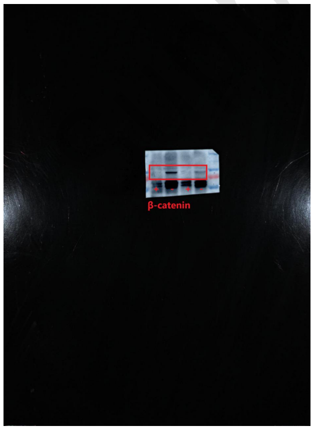

β-catenin

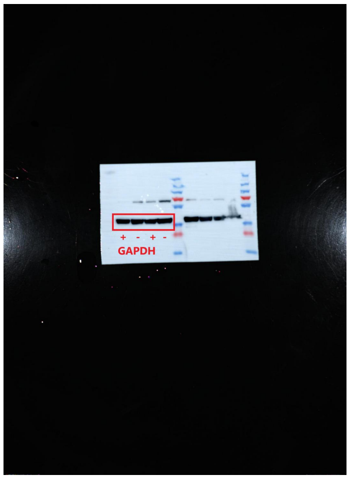

GAPDH

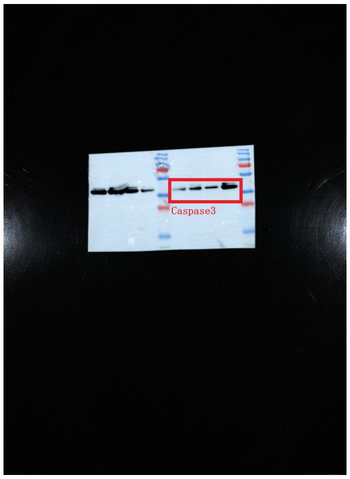

Caspase3

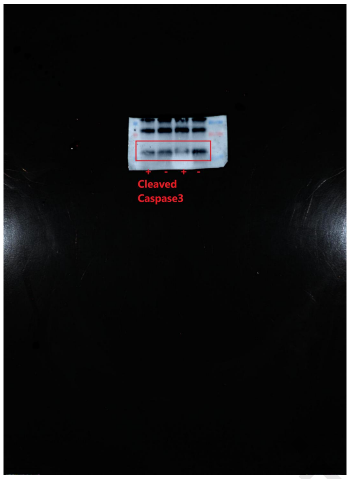

Cleaved Caspase3

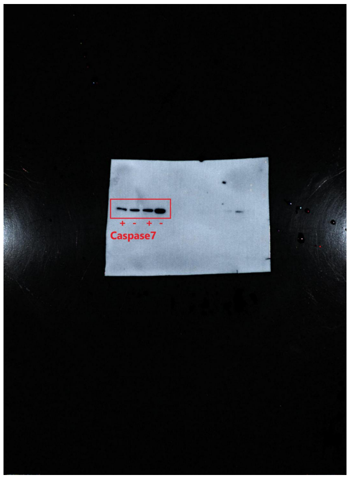

Caspase7

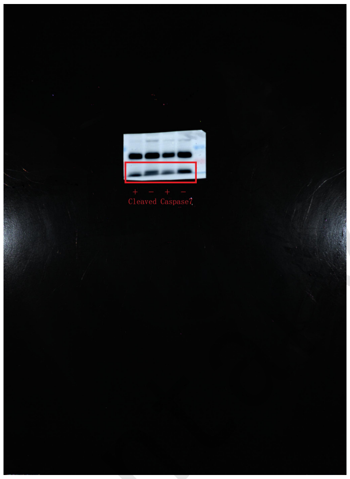

Cleaved Caspase7

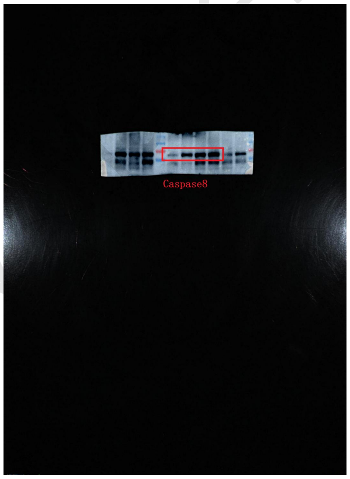

Caspase8

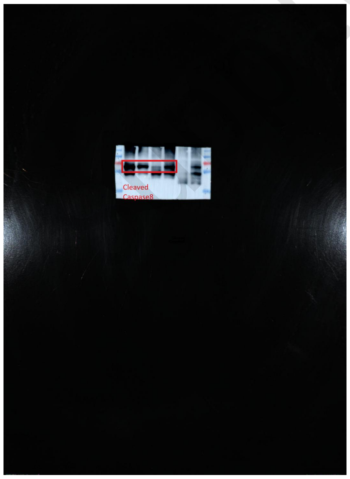

Cleaved Caspase8

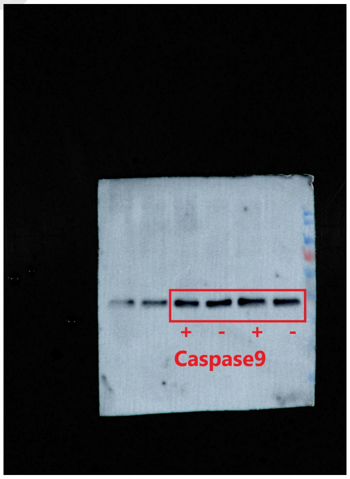

Caspase9

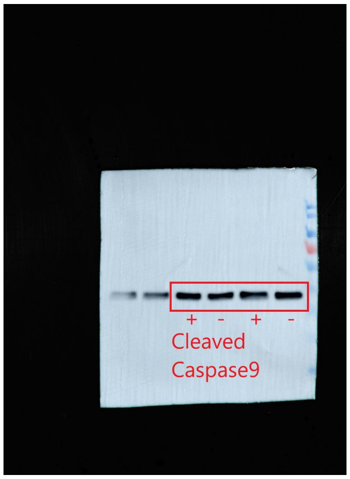

Cleaved Caspase9

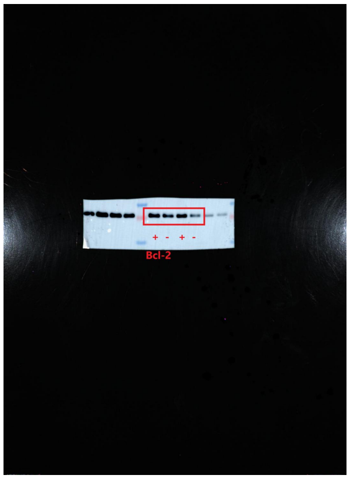

Bcl-2

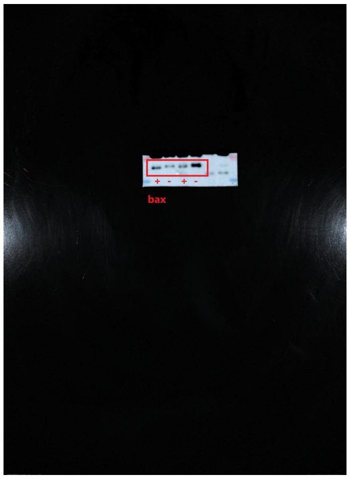

Bax

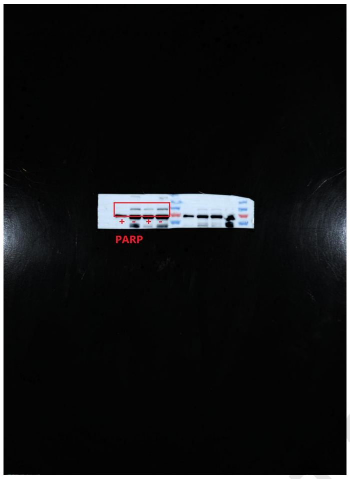

PARP

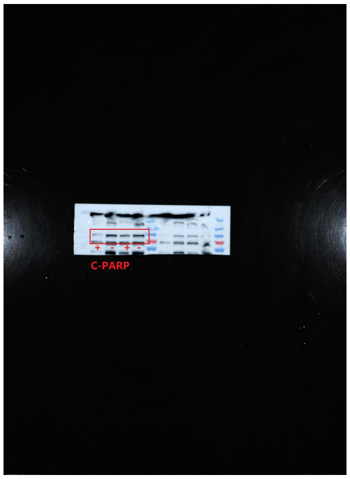

Cleaved PARP

Supplementary Materials
